# Assessment of Past Dioxin Emissions from Waste Incineration Plants Based on Archive Studies and Process Modeling: A New Methodological Tool

**DOI:** 10.1007/s00244-025-01150-9

**Published:** 2025-09-08

**Authors:** Xiaocheng Zhang, Alexis de Aragao, Fabien Moll-François, Aurélie Berthet, Florian Breider

**Affiliations:** 1 Ecole Polytechnique Fédérale de Lausanne (EPFL), School of Architecture, Civil and Environmental Engineering, 1015 Lausanne, Switzerland; 2 Ecole Polytechnique Fédérale de Lausanne (EPFL), College of Humanities, 1015 Lausanne, Switzerland; 3 Department of Occupational and Environmental Health (DSTE), Center for Primary Care and Public Health (Unisanté), University of Lausanne, 1066 Epalinges-Lausanne, Switzerland

## Abstract

Pollution from past industrial activities can remain unnoticed for years or even decades because the pollutant has only recently gained attention or been identified by measurements. Modeling the emission history of pollution is essential for estimating population exposure and apportioning potential liability among stakeholders. This paper proposes a novel approach for reconstructing the history of polychlorinated dibenzo-*p*-dioxin (PCDD) and polychlorinated dibenzofuran (PCDF) pollution from municipal solid waste incinerators (MSWIs) with unknown past emissions. The proposed methodology relies on the search for technical and operational data on the pollution source in archives, the extraction of representative data from the scientific literature, and the use of kinetic models of the formation and decomposition of PCDD/Fs within combustion chambers. This new methodological tool allows to estimate any MSWI’s stack emission and relative profile of seventeen PCDD/F congeners over time. The approach is validated through a case study of an MSWI in Switzerland. The modeled congener profile achieved a Pearson correlation coefficient of 0.98 with measurements in fly ash washwater. Additionally, the simulated soil quantity (1,115–1,419 gTEQ _WHO-2005_ or 1,283–1,698 gTEQ_WHO-2022_) agrees in order of magnitude with the estimated quantity from measurements (371 gTEQ _WHO-2005_ or 425 gTEQ _WHO-2022_).

**Graphical Abstract:**

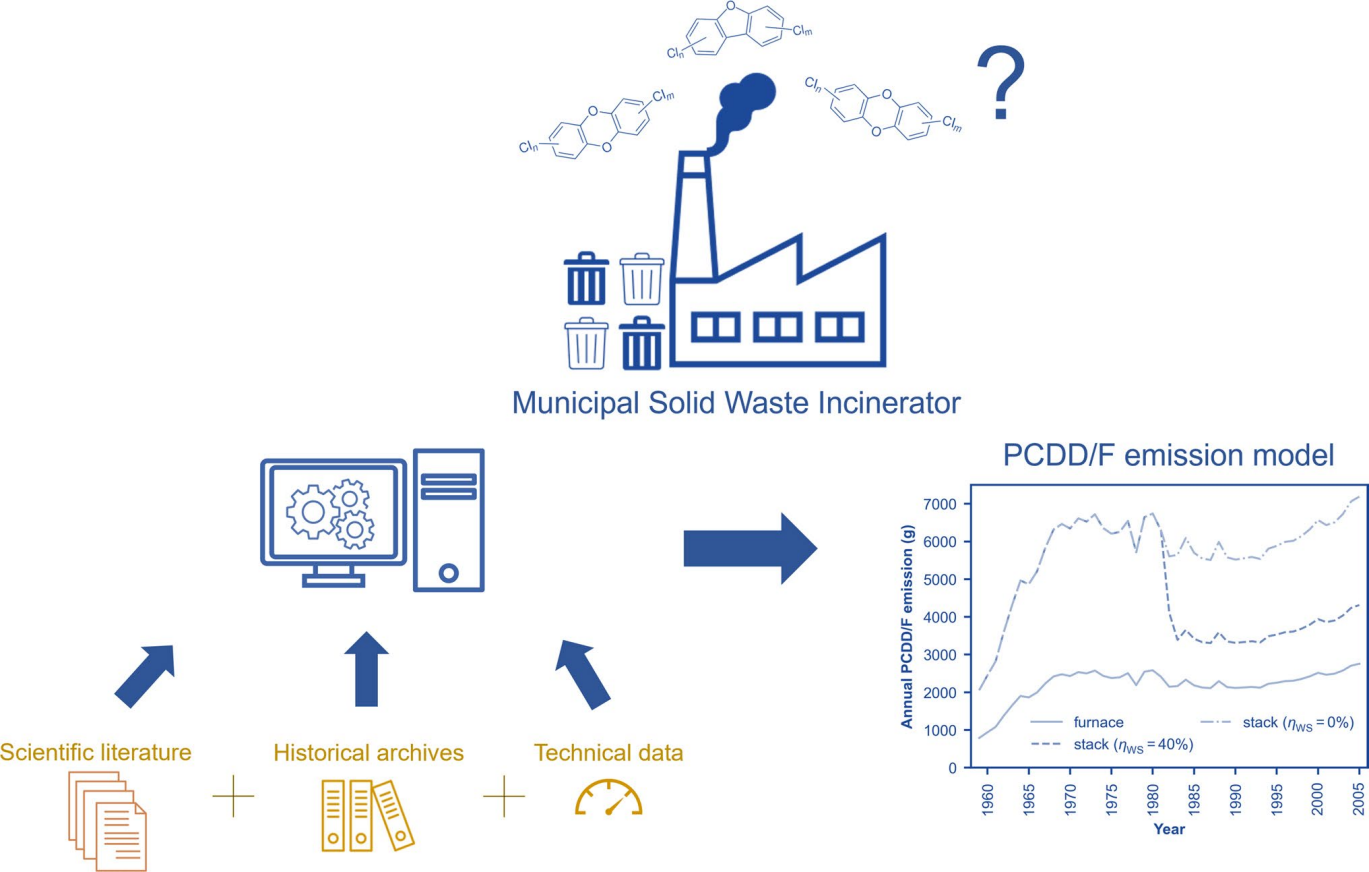

**Supplementary Information:**

The online version contains supplementary material available at 10.1007/s00244-025-01150-9.

Polychlorinated dibenzo-*p*-dioxins (PCDDs) and furans (PCDFs) are two groups of ubiquitous and persistent contaminants released from industrial and natural combustion (Kanan and Samara 2018). They exist in the environment as complex mixtures of 210 possible congeners, of which 7 PCDDs and 10 PCDFs with chlorine substitution on the 2, 3, 7, and 8 positions have been identified as toxic to humans and mammals (van den Berg et al. 1998, 2006; DeVito et al. 2024). The seventeen PCDD/F congeners of toxicological relevance, as well as their toxic equivalency factors (TEFs), are displayed in Supplementary Information [WHO Toxic Equivalency Factors of 17 PCDD/Fs]. It should be noted that this study does not cover non-ortho-substituted polychlorinated biphenyls (PCBs), whose structure and mechanism of toxic action are comparable to those of PCDD/Fs.

Anthropogenic thermal processes are the most prominent source of PCDD/F emission. Specifically, waste incineration plays an important role as it contributes to 13% of the total PCDD/F emission across 86 countries as of the year 2014 (Fiedler 2016), with substantially greater contributions in earlier decades. For example, U.S. EPA inventories identified municipal waste incineration as the dominant emission source in the 1980s, with a marked decline by the 2000s due to regulatory controls (U.S. EPA 2006). Upon the release of combustion fumes, atmospheric transport and deposition then lead to the pollution of PCDD/Fs in the topsoil, where they demonstrate extreme persistence with half-lives ranging from years to several decades (Seike et al. 2007). The PCDD/F polluted soil may increase health risks to humans, not only through direct ingestion of soil, but also by the consumption of food from animals raised on the contaminated land and of cucurbitaceous vegetables cultivated in highly contaminated soil (Hülster et al. 1994; Engwall and Hjelm 2000; Zhang et al. 2009; Urbaniak et al. 2017; Vernez et al. 2023). In urban environments, municipal solid waste incinerators (MSWIs) are frequently a source of concern for local populations, especially MSWIs with a long history of operation as they often lacked continuous PCDD/F monitoring during the early years of commissioning. Even when historical time series of PCDD/F measurements at MSWIs are available, they may not be sufficient for a detailed examination of the history of soil pollution, depending on the quality and frequency of the measurements.

To compensate for the missing emission data, models are required to reconstruct the PCDD/F emission history at MSWIs. The great complexity of PCDD/F synthesis pathways in incinerators and the numerous influencing factors heightened the challenge of modeling studies. Based on the available literature, no models are proposed for deriving PCDD/F congener profile in incinerators. A number of models exist for estimating the quantity of total PCDD/F emission from MSWIs. These models are largely based on the kinetics of PCDD/F formation and decomposition and rely on process parameters such as temperature, residence time, fly ash properties, and the concentration of other chemicals (Shaub and Tsang 1983; Altwicker et al. 1990; Huang and Buekens 2001; Stanmore 2002). However, the impact of waste composition and air pollution control devices (APCDs) on PCDD/F congener profile and quantity is not addressed in these studies. These aspects were explored in a probabilistic model proposed by Koehler et al. (2011), but significant uncertainties are associated with the calculated PCDD/F emission factors due to the nature of the approach.

The objective of the current study is to develop an approach to reconstruct the history of PCDD/F pollution from MSWIs. To this end, two complementary modeling steps are successively performed. Firstly, the relative profiles of PCDD/F congeners at different periods are empirically estimated on the basis of data from similar MSWIs. Secondly, the absolute amount of PCDD/F emission over time is derived using a kinetic model. This takes into account the formation and decomposition of PCDD/Fs and various parameters such as waste composition and operating conditions of the MSWI, based on historical archives. The combination of these two modeling parts and the study of archives provides the congener-specific emissions over the entire lifetime of an MSWI under investigation.

The developed methodology is further validated in a concrete case of PCDD/F pollution from a former MSWI. The MSWI was located in Lausanne, Switzerland, and operated between 1958 and 2005. The validation analysis indicates considerable PCDD/F contamination by the MSWI, in agreement with recent soil measurements. Additionally, simulations under different scenarios are performed to quantify the impact of important parameters, including waste composition, operating conditions, and APCDs.

The tool can be used to estimate the order of magnitude of past PCDD/F emissions from MSWIs. It also provides an estimated congener profile as a fingerprint of emission characteristics. While the tool is suitable for an initial assessment of pollution severity, it is not designed for human risk analysis. The latter requires considering the fate and exposure pathways of PCDD/Fs in various matrices, while the validation case in this study focuses on soil concentrations.

## Methods

The modeling framework describes the congener profile and total emission quantity of PCDD/Fs at the stack of an MSWI of interest. The model relies on historical archives, technical data, and scientific literature as sources of data.

**Fig. 1:**
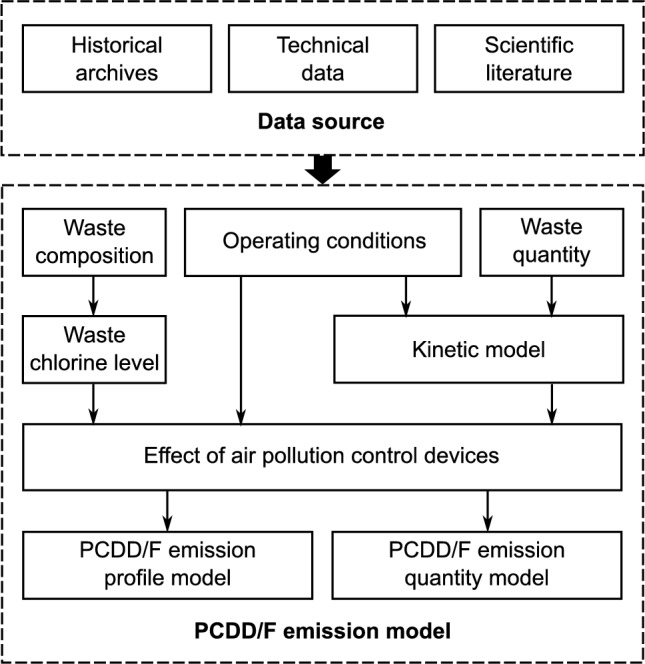
Model framework for PCDD/F congener profile and emission quantity at an MSWI stack

The model’s boundaries consist of waste quantity, waste composition, operating conditions, APCD configuration, and PCDD/F formation and decomposition during incineration (Fig. 1). The key parameters include the quantity and material composition of the incinerated waste, the operating conditions, and the APCD configuration. These data are typically obtained from archival documents.

The chlorine content in the waste stream is estimated from waste composition, and the typical congener profile corresponding to this chlorine level and operating conditions is drawn from the literature. A kinetic model, available in the literature, is employed to simulate the total amount of PCDD/Fs generated in the furnace. Finally, the effect of APCDs is considered using existing literature data from MSWIs with similar APCD configurations as references.

### Estimating PCDD/F Relative Profile

The proposed method for estimating the relative profile of the 17 toxic PCDD/F congeners at an MSWI’s stack for different time periods is empirical. It relies on profile data obtained from other incinerators that closely resemble a target MSWI at specific time intervals. Such representative data are typically available in scientific literature, as numerous studies have investigated various types of incinerators (Oh et al. 1999; Takaoka et al. 2003; Chang et al. 2004; Lin et al. 2020; Wang et al. 2022).

The degree of similarity between an incinerator and a target MSWI can be evaluated based on three factors: the chlorine content of the input waste, the APCDs in place, and the operating conditions. Indeed, previous research, employing principal component analysis (PCA), has demonstrated that the chlorine content of the input waste contributes to approximately 40% of the overall variance in relative PCDD/F profiles, while other factors such as the APCD configuration and operating conditions account for the remaining 60% (Wang et al. 2003). Therefore, when estimating the relative PCDD/F profile at an MSWI stack for a specific period, the data sources should at least strive to minimize biases associated with these three explanatory factors: chlorine content of input waste, APCDs, and operating conditions.

#### Chlorine Content of Input Waste

To estimate the average chlorine content of waste at a studied MSWI, for any undisclosed period, the following steps must be followed:

1. Ascertain the average mass composition of the waste on a per-category basis;
2. Determine the average chlorine content associated with each waste category;
3. Estimate the average chlorine content of the MSWI’s input waste for the studied time by computing a weighted average across the waste categories, applying Eq. 1:
  1 $$ {\text{Cl}}_{{\text{i}}} = \mathop \sum \limits_{{\text{j}}} {\text{Cl}}_{{\text{j}}} \cdot n_{{\text{i,j}}} $$

where ${\text{Cl}}_{{\text{i}}}$ represents the average chlorine content (on a per-mass basis) of the MSWI’s input waste during the period $i$, ${\text{Cl}}_{{\text{j}}}$ denotes the average chlorine content (on a per-mass basis) associated with the waste category $j$, and $n_{{\text{i,j}}}$ are the mass share of the waste type $j$ during the period $i$.

A procedure for estimating the weight fraction of chlorine in the waste from categorical waste composition is presented in Supplementary Information [Waste Element Composition]. This involves a search for data on the source of pollution, which may typically be found in certain archives. The estimated chlorine content should be then compared against a critical threshold of 0.8–1.1%, as this range marks a distinction between two types of PCDD/F stack profiles (Wang et al. 2003).

#### Air Pollution Control Devices (APCDs)

Ensuring representativeness of reference incinerators for a target MSWI demands a matching in APCD configuration, including both sequence and equipment. This suggests having a solid understanding of the APCD configuration of a target MSWI for the studied period. Technical documents are likely to be found in archives.

Empirical data can cover the whole APCD chain or only part of it. When no reference incinerator fits the entire APCD array’s configuration, data from different sources are averaged, or the evolution of the profile throughout the treatment process is studied. More specifically, data on the efficiency of every APC chain or device in altering the amount of each PCDD/F congener before and after treatment, for each investigated period, are needed. Additionally, the initial PCDD/F profile (i.e., post-combustion profile, before any treatment) is required. Leveraging this initial profile and APCD efficiencies subsequently enables the systematic propagation of the PCDD/F relative profile from the furnace through the post-combustion zone up to the stack. The removal efficiency of each congener $i$ by the APC chain or device $j$ is defined according to Eq. 2:

2 $$ \eta_{i,j} = 1 - \frac{{C_{{{\text{out}},{ }i}} }}{{C_{{{\text{in}},{ }i}} }} $$

where $C_{{{\text{in}},i}}$ and $C_{{{\text{out}},i}}$ are the mass concentrations of congener $i$ at the inlet and outlet of the APC chain or device $j$, respectively. The evolution of the congener relative profile can then be modeled considering Eq. 3:

3 $$ R_{{i,j_{{{\text{out}}}} }} = R_{{i,j_{{{\text{in}}}} }} \cdot \frac{{\left( {1 - \eta_{i,j} } \right) }}{{\mathop \sum \nolimits_{k}^{17} \left[ {\left( {1 - \eta_{k,j} } \right) \cdot R_{{k,j_{{{\text{in}}}} }} } \right] }} $$

where $$\eta_{{\text{i,j}}}$$ represents the mass–concentration-based efficiency of the APC chain or device $$j $$ on the congener $$i$$, and $$R_{{{\text{i,j}}_{{\text{in/out}}} }}$$ is the mass fraction of the congener $$i$$ over the 17 relevant PCDD/F congeners, respectively, at entrance (in) and exit (out) of the APC chain or device $$j$$.

For cases where removal efficiencies are presented in terms of congener mass fractions, or there is an expected efficiency discrepancy between a target MSWI and a reference incinerator that necessitates adjustment, alternative methods are available in Supplementary Information [APCD Efficiency Adjustment].

#### Operating Conditions

When assessing the similarity between reference incinerators and a target MSWI, temperature is identified as the primary variable influencing the formation of PCDD/Fs within incinerators, as reported in a previous study (McKay 2002). Additionally, some secondary operating factors, such as waste composition, oxygen supply, and shutdown/start-up phases, are shown to impact the generation of PCDD/F congeners in incinerators (Tejima et al. 2007; Zhang et al. 2008; Li et al. 2019). These secondary factors are, however, not considered in this study due to limited insights regarding their influence on PCDD/F formation, their variability, and the challenges associated with deriving accurate data.

For the temperature factor, the efficient elimination of PCDD/Fs found in waste materials primarily takes place within the furnace, following the “3 T + E” principle. The latter implies a temperature exceeding 850 °C, a residence time of more than 2 s, ensuring adequate turbulence, and having an excess of air (McKay 2002). However, PCDD/Fs are regenerated in the post-combustion zone as the flue gas cools down, through the following mechanisms (for more details, please refer to Supplementary Information [PCDD/F Formation Mechanism]):

1. Homogeneous synthesis (500–800 °C).
2. Heterogeneous de novo synthesis (200–400 °C).
3. Heterogeneous precursor synthesis (200–400 °C).

The temperature decrease in the post-combustion zone has thus an impact on the relative PCDD/F profile, given that PCDD/F synthesis rates are responsive to temperature levels and are congener specific. Estimating the PCDD/F profile at the stack of a target MSWI therefore requires that the reference incinerators, which serve as data sources, show close resemblance in terms of temperature conditions at crucial points, including the furnace, the upstream post-combustion zone, and the APCDs of interest, both at inlets and outlets. The need for similarity in temperature levels is especially critical for APCD profile data where APCDs operate within the temperature range of 200–400 °C. This is because these temperature conditions enable heterogeneous synthesis of PCDD/F congeners, which exhibit a sensitivity to temperature fluctuations, as mentioned earlier.

### Estimating PCDD/F Emission Quantity

Estimating the total stack emissions of the seventeen PCDD/F congeners relies on the use of a kinetic model introduced by Palmer et al. (2021). The model was fitted and evaluated against published experimental data with a consistent analytical procedure and various types of waste. It computes the generation of a specific congener in an MSWI’s furnace and thus allows estimating the total amount of PCDD/F generation using the proportion of the specific congener from the relative profile estimation (see Sect. “Estimating PCDD/F Relative profile”). Additionally, the influence of APCDs on the calculated furnace’s emission is considered by using data from existing literature studies. This analysis allows for deriving the total quantity of PCDD/F emissions at the stack of a target MSWI.

The two-step model proposed by Palmer et al. (2021) to estimate the quantity of PCDD/F emissions in an incinerator considers the first-order kinetics of formation and decomposition of PCDD/Fs with congener-specific rate constants, as outlined in Eq. 4 and Eq. 5:

4 $$ C + a{\text{O}}_{{2}} \to ^{{k_{{\text{1,i}}} }} b{\text{CO}} + c{\text{CO}}_{{2}} + d\left[ {{\text{aromatics}}} \right] + f_{{\text{i}}} \left[ {\text{PCDD/Fs}} \right]_{{\text{i}}} $$

5 $$ \left[ {PCDD/Fs} \right]_{{\text{i}}} + {\text{O}}_{{2}} \to ^{{k_{2} }} {\text{other products}} $$

where $C$ represents the carbonaceous part of the waste (g carbon/g waste), $f_{{\text{i}}}$ represents the yield of each congener (g of congener/g of decomposed carbon), $k_{{\text{1, i}}}$ denotes the formation rate constant of each congener (s^−1^), and $k_{2}$ denotes the decomposition rate constant (s^−1^).

Several influencing factors on the kinetics are considered in the model, including waste chlorine and metal content, combustion air oxygen content, and combustion temperature. The formula quantifying the effect of each factor is detailed in Supplementary Information [Emission Quantity Model]. The optimized estimates of kinetic constants are provided by Palmer et al. (2021) for three congeners: 2,3,7,8-TCDF, OCDF, and 1,2,3,6,7,8-HxCDD. The congener exhibiting the greatest relative share in the congener profile at the MSWI under investigation (as computed from Sect. “Estimating PCDD/F Relative profile”) is selected, therefore reducing vulnerability to uncertainties in parameter estimates.

Given the kinetic model and parameters, the quantity of generated PCDD/Fs in the furnace at a specific residence time can be obtained from the following analytical solution, written as Eq. 6:

6 $$ \left[ {\text{PCDD/Fs}} \right]_{{\text{i}}} = \frac{{k_{{\text{1,i}}} \cdot E_{{{\text{Cl}}}} \cdot E_{{{\text{metal}}}} }}{{k_{2} }} \cdot \left( {1 - {\text{exp}}\left( { - k_{2} \cdot \lambda_{{{\text{oxygen}}}} \cdot t} \right)} \right) $$

where $\left[ {\text{PCDD/Fs}} \right]_{{\text{i}}}$ is the generated amount of congener $i$ per unit mass of incinerated waste, $E_{{{\text{Cl}}}}$ and $E_{{{\text{metal}}}}$ denote the weight fraction of chlorine and metal in the waste, respectively, $\lambda_{{{\text{oxygen}}}}$ is the oxygen ratio, and $t$ is the flue gas residence time in the furnace.

### Validation Case

The approach presented in this paper is validated by comparing the model-simulated emissions of PCDD/F congeners with the actual soil concentrations of these pollutants in the vicinity of the Vallon MSWI. The facility was situated in Lausanne, Switzerland, and was operational from 1958 to 2005. Significant PCDD/F pollution was discovered in Lausanne soil in 2020, and the Vallon MSWI was identified as the source. For a detailed account of the Vallon MSWI’s historical, technical and operational backgrounds, please refer to Supplementary Information [Vallon History], [Vallon Technical Aspects], [Vallon History of Waste Properties and Combustion Quality]. The results of the validation analysis are presented and discussed in Sect. “Results and discussion”.

### Sensitivity Analysis

On the basis of the validation case, a sensitivity analysis is performed in order to identify the most relevant parameters to the emission quantity modeling results. The sensitivity analysis is conducted using Latin Hypercube Sampling, with input parameters drawn from truncated normal distributions. The result of the analysis is presented as contribution factors, representing the uncertainty in model output attributed to each input parameter. The contribution factor is computed as the normalized squared rank correlation:

7 $$ k_{{\text{i}}} = \frac{{\rho_{{\text{i}}}^{2} }}{{\mathop \sum \nolimits_{{\text{i}}} \rho_{{\text{i}}}^{2} }} $$

where $\rho_{{\text{i}}}$ is the Spearman rank correlation coefficient calculated between each input and the model output after rank transformation. The detailed methodology is demonstrated in Supplementary Information [Sensitivity Analysis].

### Data Source

As part of the present study, a large number of archives and printed sources are collected. They are used to feed the developed model with data specific to the validation case, i.e., the PCDD/F pollution caused by the Vallon MSWI. In particular, archival documents are needed to characterize the pollution source in terms of operational and technical aspects, as well as to provide information on the related soil pollution, be its extent or its magnitude.

The archives and printed sources used for this work focus on the 1952–2005 period. They can be divided into three categories:

1. Internal archives of the former Vallon MSWI. These encompass all plant management documents and are currently held by Tridel S.A. in Lausanne. They include correspondence, meeting minutes, internal memos, and technical logs tracing the Vallon MSWI operations.
2. Archives of the municipal and cantonal services in charge of managing and controlling the Vallon MSWI in the past. These cover archives of the City of Lausanne, archives of the Canton de Vaud, and internal archives of the Vaud State Department of the Environment (DGE-Vaud). Among them are monitoring records. For the purposes of the Vallon validation case, such documents have been exploited, including the analysis campaigns on household waste composition, the spot measurements of emissions from the MSWI stack (other than PCDD/F), and the measurements of PCDD/F concentrations in Lausanne wastewater in the 1990s.
3. Documents released by professional associations. Typically, these are written by experts in their field and address very specific topics and issues. They often take the form of conference papers or professional journal articles. To support the Vallon validation case, such documents have been gathered, for example, technical publications on the Vallon MSWI or articles on pollution around the incinerator published in the 1970s and 1980s.

In addition, data from the scientific literature are used and primarily serve emission profile modeling. The search terms include variations in “PCDD,” “PCDF,” and “dioxin,” combined with terms related to specific APCD employed by the Vallon MSWI, such as “electrostatic precipitator” and “wet scrubber.” The retrieved papers are screened for the similarity of the incinerators they study to the target MSWI. Additionally, two articles are used to establish the mapping between waste categorical composition and waste chlorine level. Consequently, the following studies are integrated in the validation analysis:

- Emission profile model (Takaoka et al. 2003; Chang et al. 2004).
- Waste chlorine level (Themelis 2010; Liu et al. 2018).

## Results and discussion

### Analysis on Data Source

#### 3.1.1. Chlorine Content

The estimation of chlorine content in the input waste at Vallon is performed by segmenting the operational period of the MSWI, spanning from 1958 to 2005, into five discrete periods. The estimated values range from 0.3 to 0.7% by weight (refer to Table 1) with an upward trajectory. The trend can be attributed to the growing proportion of plastics in household waste as an important chlorine source. Themelis et al. (2010) reported in their study that plastics contribute to nearly half of the chlorine content in municipal waste, despite being only 10% by weight.

**Table 1:** Estimated time evolution of the waste chlorine content at the Vallon MSWI

Period	Cl content (wt %)
1958–1965	0.3
1965–1975	0.4
1975–1985	0.5
1985–1995	0.5
1995–2005	0.7

Vallon waste composition is estimated on the basis of five sources: the United States Environmental Protection Agency (EPA) national overview on materials, wastes and recycling for the year 1960, an analysis of the contents of Zurich’s garbage cans for the year 1969 reported by Desbaumes and Imhoff (1971), a waste survey carried out in Lausanne in 1982 and reported by Voelgyi (1985), a survey of the contents of Lausanne’s garbage cans in 1990 found in the Lausanne Archives, and the national analysis of waste composition carried out by the former Swiss environment office SAEFL for the years 2000/2001 (SAEFL 2003).

#### 3.1.2. APCD Configuration

Based on technical documents found in archives, two main periods regarding APCDs can be distinguished at Vallon MSWI: between 1958 and 1982, when only electrostatic precipitators (ESP) were implemented, and between 1982 and 2005, when electrostatic precipitators followed by wet scrubbers were operating (ESP + WS).

#### 3.1.3. Operating Conditions

**Fig. 2:**
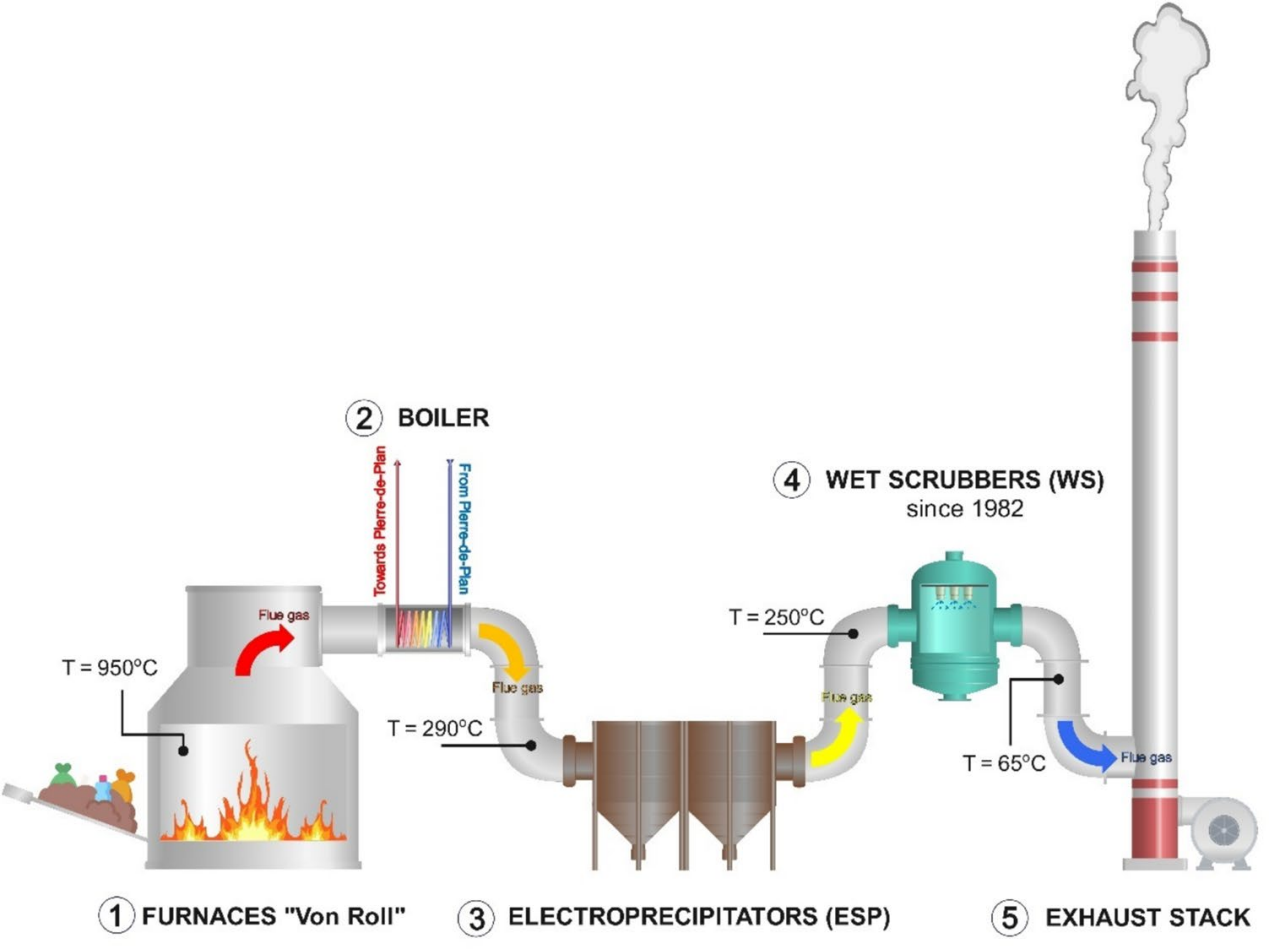
Main technical and operational aspects of the Vallon MSWI

According to archival technical documents, the Vallon MSWI featured two “Von Roll” furnaces functioning from October 6, 1958, to December 29, 2005 (① in Fig. 2). The incineration chambers maintained a typical temperature range of 950 ± 50 °C. Subsequently, hot flue gases exited the furnaces, entered the afterburner area, and underwent cooling to 280–300°C through boiler heat exchange (② in Fig. 2), allowing for significant PCDD/F synthesis. The flue gas then passed through the ESP for particle removal (③ in Fig. 2). While particulate PCDD/Fs were probably slightly removed in Vallon’s ESP, a significant amount was also likely produced there, due to favorable temperatures. After the ESP treatment, the flue gas temperature typically ranged between 250 and 300 °C. Post-1982, flue gas was further directed to the WS before exhaust (④ in Fig. 2). The WS removed dust particles through wetting, and within Vallon’s WS, gaseous PCDD/Fs were probably partially removed through condensation. However, limited elimination of particulate PCDD/Fs is expected due to the low solubility of PCDD/F species. Noteworthy, the temperature conditions inside the WS partially overlapped PCDD/F synthesis windows. Finally, flue gas was discharged into the atmosphere through the 80-m-high stack at a low temperature, typically in the range of 60–65 °C (⑤ in Fig. 2).

### PCDD/F Relative Profile

To estimate the PCDD/F relative profile at the Vallon MSWI’s stack, profile data from similar incinerators are sought in the literature. The evaluation of similarity is based on the three previously described (Sect. "Estimating PCDD/F Relative profile") and quantified (Sect. "Analysis on Data Source") explanatory factors: chlorine content of the waste input, APCD configuration, and operating conditions.

Based on the estimates of the explanatory variables, two periods (also called scenarios) have been established for reconstructing the PCDD/F relative profile at the Vallon MSWI’s stack. Scenario n°1 (1958–1982) corresponds to the period when the ESP was the sole flue gas control system in the post-combustion area. Scenario n°2 (1982–2005) pertains to the next period, during which WS were introduced to complement the ESP. Regarding the chlorine content of waste input and operating conditions, these factors can be considered uniform across both of these periods. An extensive literature search has next been carried out to find representative profile data for Vallon MSWI concerning these two scenarios.

For scenario n°1 (1958–1982), only the profile measurements carried out by Chang et al. (2004) at an MSWI in Taiwan are considered applicable to the Vallon case. Indeed, the similarities between the Vallon MSWI and this Taiwanese incinerator are multiple. Both processes primarily solid household waste and seem to have chlorine levels below the 0.8–1.1% threshold. The temperature of the furnace in Taiwan is between 850 and 1050 °C, close to the 900–1000 °C declared at Vallon. In addition, the pollution control system after the boiler consists of an ESP followed by a WS in Taiwan, exactly as at Vallon. However, the ESP inlet temperature of the flue gas is lower in Taiwan (i.e., 234 °C instead of 280–300 °C at Vallon). Higher ESP inlet temperature implies higher vapor pressure of PCDD/Fs, leading to a greater percentage in the gas phase and decreased global removal efficiency because the ESP is only effective for particle-bound PCDD/Fs. The removal efficiencies of the ESP on congener fractions, as measured by Chang et al. (2004) for the particulate fraction, are therefore corrected to take into account the higher temperature at Vallon. The applied adjustment method is presented in Supplementary Information [Vallon Congener Profile Estimation]. Table 2 shows the final estimates for Vallon ESP. In column a), the ratios of the 17 congeners to the total PCDD/F concentration before the ESP are indicated, as well as the gas/particle phase distribution. Column b) refers to the situation after the ESP. Column c) lists the absolute removal efficiencies of the ESP on the PCDD/F congeners, also with phase distinction.

**Table 2:** PCDD/F profile before and after the ESP, along with the ESP (concentration-based) removal efficiencies, categorized by gaseous (G.), particulate (P.), and total (G. + P.) phases, as estimated for the Vallon MSWI

Congener	a) Before ESP (% PCDD/F conc.)			b) After ESP (% PCDD/F conc.)			c) ESP removal efficiency (% conc.)		
	G	P	G. + P	G	P	G. + P	G	P	G. + P
2,3,7,8-TeCDD	0.1	0.1	0.2	0.1	0.0	0.1	− 173	54	− 79
1,2,3,7,8-PeCDD	0.4	0.3	0.7	0.9	0.1	1.0	− 479	1	− 279
1,2,3,4,7,8-HxCDD	0.4	0.4	0.8	1.1	0.2	1.3	− 631	− 29	− 334
1,2,3,6,7,8-HxCDD	0.8	0.7	1.5	2.9	0.5	3.3	− 857	− 71	− 479
1,2,3,7,8,9-HxCDD	0.5	0.5	1.0	1.4	0.3	1.7	− 610	− 37	− 318
1,2,3,4,6,7,8-HpCDD	3.8	4.6	8.4	12.0	2.6	14.6	− 719	− 46	− 352
OCDD	11.1	14.9	26.0	20.4	5.1	25.5	− 380	11	− 156
2,3,7,8-TeCDF	0.6	0.4	1.0	0.8	0.1	1.0	− 265	27	− 141
1,2,3,7,8-PeCDF	0.9	0.8	1.8	1.7	0.3	1.9	− 363	15	− 186
2,3,4,7,8-PeCDF	1.8	1.7	3.6	3.7	0.7	4.5	− 429	− 11	− 226
1,2,3,4,7,8-HxCDF	1.3	1.4	2.7	2.9	0.5	3.4	− 484	0	− 236
1,2,3,6,7,8-HxCDF	1.5	2.0	3.6	3.4	0.8	4.2	− 470	− 3	− 205
1,2,3,7,8,9-HxCDF	0.1	0.2	0.3	0.3	0.1	0.4	− 403	− 17	− 205
2,3,4,6,7,8-HxCDF	3.7	3.8	7.5	5.9	1.6	7.5	− 320	− 8	− 161
1,2,3,4,6,7,8-HpCDF	8.3	9.4	17.7	12.1	3.0	15.1	− 283	18	− 123
1,2,3,4,7,8,9-HpCDF	1.2	1.6	2.7	1.8	0.6	2.4	− 312	1	− 131
OCDF	8.3	12.4	20.7	8.9	3.4	12.3	− 180	29	− 55
Σ PCDDs	17	21	39	39	9	47	− 491	− 6	− 221
Σ PCDFs	28	34	61	42	11	53	− 291	15	− 123
Σ PCDDs + PCDFs	45	55	100	80	20	100	− 367	7	− 161

As one can see, the passage through the ESP notably affects the congener gas/particle phase distribution, with a sharp decrease in the particulate fraction (55% before the ESP, 20% after). The total (gaseous + particulate) congener pattern is also affected, with significant reductions in highly chlorinated fractions such as OCDF, 1,2,3,4,6,7,8-HpCDF and to a lesser extent OCDD. According to Chang et al. (2004), the gas/particle phase distribution depends on the vapor pressure of each congener, and thus on the temperature. As temperature decreases in the after-combustion area, so do the vapor pressures, and more congeners are adsorbed onto the particles. The latter are susceptible to removal by the ESP, while gaseous PCDD/Fs mostly pass through. Highly chlorinated congeners as well as PCDFs have a lower vapor pressure. This is the reason why 1,2,3,4,6,7,8-HpCDF, and OCDF are more likely to condense on the particles and undergo higher removal. Interestingly, the conditions inside the ESP correspond to the temperature window of the de novo synthesis (200–400 °C), and many gaseous PCDD/Fs are likely to form in this after-combustion area. This is confirmed by the total PCDD/F removal efficiency of the ESP, which is estimated to be strongly negative (− 161% on a PCDD/F concentration basis).

Regarding scenario n°2 (1982–2005), integrating the effect of the WS, only data from Takaoka et al. (2003) and once more, Chang et al. (2004), are considered applicable. The incinerators studied in these two papers closely match the Vallon MSWI concerning the three criteria of interest, including chlorine content, the presence of APCDs, and operating conditions. The incinerators investigated by Takaoka et al. (2003) consist of two MSWIs (named MSWI-A and MSWI-B) commissioned, respectively, in 1980 and 1985, which cover the period of operation of the Vallon. In addition, MSWI-A and MSWI-B are only equipped with an ESP before the WS, exactly as at Vallon. It should be noted that MSWI-A differs from MSWI-B in several respects regarding the WS. The scrubbing water circulation rate in MSWI-A is higher, and the salt concentrations in the scrubbing water are maintained at 3% and 8% in MSWI-A and MSWI-B, respectively.

Two notable insights are gained from Takaoka et al. (2003) and Chang et al. (2004)’s figures for the 1982–2005 period. First, the installation of a WS at the Vallon MSWI is likely to have significantly lowered the fraction of PCDD/Fs in the gas phase relative to particles, i.e., 65% decrease in the gaseous fraction reported by Chang et al. (2004) for the reference MSWI. This reduction effect can be attributed to the dual actions of condensation within the WS and congener capture in the WS solution. Second, the WS removal efficiency was probably higher for congeners with lower chlorination levels or belonging to PCDDs. This can be explained by the fact that these compounds are characterized by higher vapor pressure and are thus more prevalent in the gas phase before treatment, which renders them more susceptible to removal by the WS. Additionally, less chlorinated compounds could be more soluble and more reactive to the scrubbing solution.

**Fig. 3:**
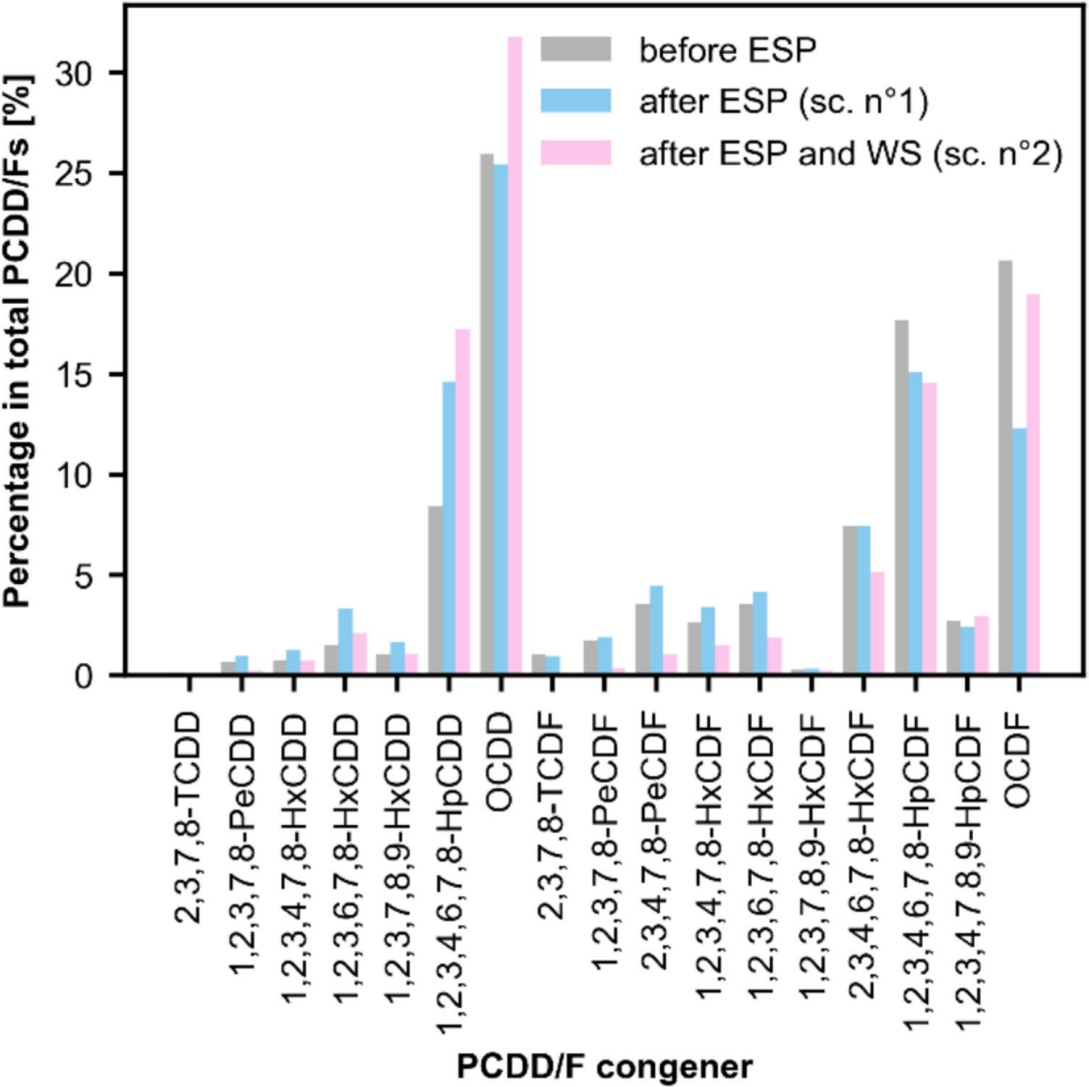
PCDD/F total (gaseous + particulate) congener profile before the ESP (gray bars), after the ESP (scenario n°1, blue bars), and after the ESP and WS (scenario n°2, pink bars), as estimated for the Vallon MSWI

Figure 3 and Supplementary information [Vallon Congener Profile Estimation] summarize the PCDD/F congener profiles for the total phase (gas + particulate) before the ESP, after the ESP (scenario n°1), and after the WS (scenario n°2) as estimated for the Vallon MSWI. The scenario n°1, after the ESP, is estimated to be the situation at the stack between 1958 and 1982, and scenario n°2, after the WS, is estimated to be the situation at the stack between 1982 and 2005.

### PCDD/F Emission Quantity

**Fig. 4:**
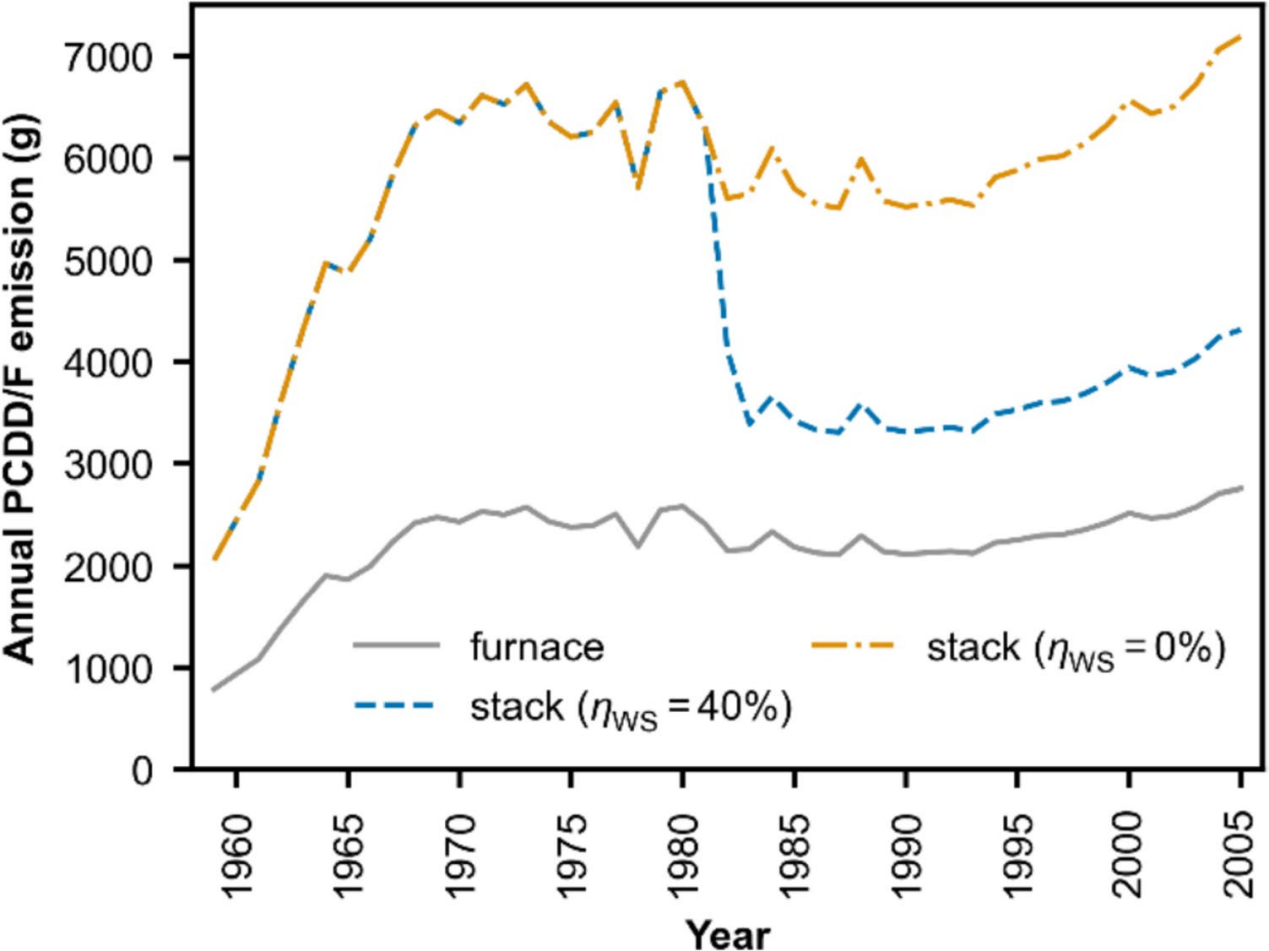
Estimated annual production of PCDD/Fs in the furnace and emission at the stack of Vallon MSWI over the period 1958–2005, with two cases of WS removal efficiency (${\eta }_{\text{WS}}$). The WS was installed in year 1982

The total quantity of the 17 PCDD/F congeners generated in the furnace is shown in Fig. 4. A description of the input parameters used in the simulation is provided in Supplementary Information [Emission Quantity Model]. The simulation with the kinetic model is performed for the congener OCDF. To obtain the total emission quantity, the amount of OCDF generated in the furnace is divided by a factor of 20.7%, which represents the estimated mass fraction of gas and particulate OCDF in the total PCDD/F before APCD processing (Table 2). The OCDF congener is selected because of its relatively large share in the congener profile, and it is thus less prone to uncertainty in the profile estimation.

The effect of APCDs on the flue gas concentration of PCDD/Fs is considered in the aforementioned scenarios: n°1 with the ESP alone (1958–1982) and n°2 with the ESP and WS (1982–2005). The temperature window in the ESP is favorable for PCDD/F formation, leading to an estimated removal efficiency for total PCDD/Fs of − 161% (Table 2), indicating that PCDD/Fs are generated rather than eliminated during this process. For a discussion of the mass balance scheme in the wet scrubber, please refer to Supplementary Information [Vallon Wet Scrubber Mass Balance].

Current knowledge of Vallon WS does not allow a precise estimate of its removal efficiency on PCCD/Fs. However, two sources provide valuable information. First, an article by Ruegg and Sigg from their time at Von Roll suggests a very low efficiency for elementary wet scrubbers using a mechanism similar to Vallon’s, citing the low solubility of PCDD/Fs in water (Ruegg and Sigg 1992). In addition, Ruegg and Sigg’s article proposes improvement methods to achieve 50% efficiency with the WS system, suggesting that the efficiency of elementary WS is significantly lower. Secondly, a report by Moll-François et al. (2024) mentions two measurements carried out in 2001 at the Vidy WWTP, Lausanne, using a LAB wet scrubber without denitrification. The measurements showed positive (75%) and then negative (− 44%) efficiencies for PCDD/Fs. The average, although not meaningful with only two measurements, is 15%. It is important to note here that extrapolating the results of the Vidy WWTP to the context of the Vallon MSWI is not straightforward, given the significant differences in technical and operational parameters. For example, the Vidy plant only burns sewage sludge, unlike the Vallon one.

Nevertheless, in view of this limited knowledge, PCDD/F emissions from the stack are estimated for two scenarios, representing the lower and upper limits of a reasonable efficiency range: (1) assuming a 0% WS removal efficiency and (2) assuming a 40% WS removal efficiency. It should be emphasized, however, that this 0 − 40% range is only a modeling scenario derived from a limited set of observations and assertions, and does not fully reflect the reality of the Vallon MSWI context. Vallon could well have experienced higher WS efficiencies, or even negative efficiencies.

Figure 4 shows the estimated annual PCDD/F production in the furnace and emissions at the stack of the Vallon MSWI over the entire operating period. The generated amount in the furnace demonstrated a rapid increase during the first 10 years of operation, mostly due to the rise in annual incinerated waste amount. It then gradually decreased from 1970 to 1990, coinciding with declines in both incinerated waste amount and metal content. The subsequent gradual increase from 1990 to 2005 corresponds with increases in chlorine and metal content. Chlorine acts as a key precursor in PCDD/F formation, while metal species are known to catalyze their synthesis through de novo mechanisms. On the other hand, the installation of the WS in 1982 could have resulted in a sharp drop in stack emissions. Still, there exists a large uncertainty with the mass balance of PCDD/Fs in the WS that requires further research.

### Validation Analysis

To validate the present methodology, model-derived estimates for the Vallon case are compared with soil and water measurements carried out in the surroundings of the Vallon MSWI. Validation approaches that rely on historical trends recorded by monitoring networks or embedded in soil or sediment cores are unavailable, as the PCDD/F contamination from the Vallon MSWI was only identified in 2020, and had not been the subject of prior analysis.

**Fig. 5:**
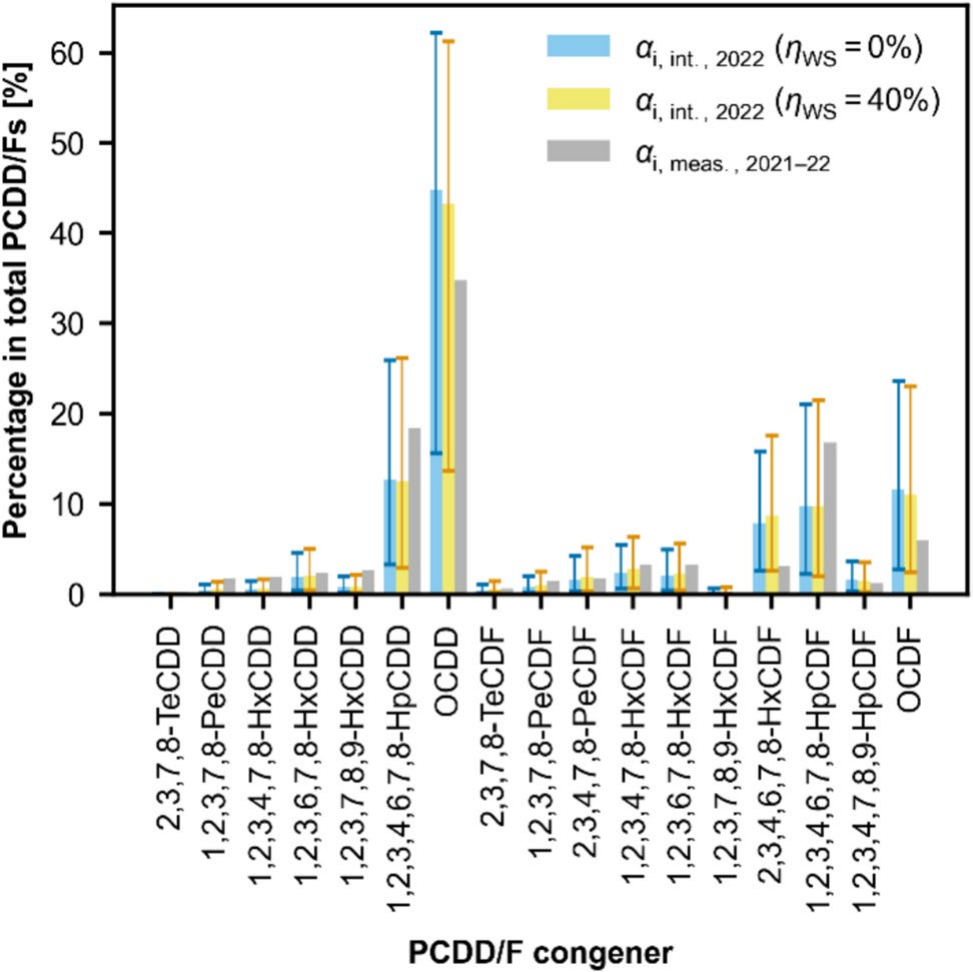
Model-derived soil profile for 2022 (blue and yellow bars) and measurement-derived soil profile for 2021–2022 (gray bars), referring to the Vallon MSWI pollution case. Soil profile simulated under two WS efficiency scenarios for PCDD/F total emissions (${\eta }_{\text{WS}}$ = 0%, blue bars and ${\eta }_{\text{WS}}$ = 40%, yellow bars). Uncertainty intervals represent the range between the 2.5% and 97.5% quantiles of the congener fractions, obtained from 1,000,000 random samples of the PCDD/F congener’s soil half-lives, varied from − 50% to + 100% of the reference values

Three validation methods are employed:

1. The first validation method involves comparing the congener distribution in the particulate phase at the ESP predicted by the model with that measured at the Vallon MSWI in 1996. The measurements were taken in the washwater of the ESP’s ash, not in exhaust gas samples. It turns out that the correlation between the congener fractions estimated in the fly ash at the ESP and the fractions measured in the ESP washwater in 1996 is excellent with a correlation coefficient score of 0.98.
2. The second, more advanced validation method begins by calculating the Vallon’s stack emissions of each congener based on the profile and quantity model. Emissions are further processed for comparison with soil measurements conducted in the Lausanne region in 2021 and 2022. The processing includes an adjustment for soil sorption phenomena, based on information from Wallenhorst (1996), and a correction for degradation mechanisms, derived per information from two papers studying PCDD/Fs in a Japanese rice field soil and a sludge-amended soil (McLachlan et al. 1996; Seike et al. 2007). In the absence of more suitable data, the congener half-lives derived from McLachlan et al. and Seike et al. are considered as the most transposable to the Lausanne soil context. Nevertheless, the departure in soil conditions constitutes an important limitation of the validation analysis. To address this, the congener half-lives are individually varied by -50% to + 100% of their nominal values to assess the sensitivity of the resulting profiles. It is found that the observed congener profiles in Lausanne align well with the modeled profiles (see Fig. 5).
3. The third validation method consists of comparing the residual amount of PCDD/Fs present in Lausanne soils in 2022, as derived from the profile and quantity model and from the spatial interpolation of the 2021–2022 soil measurements. Quantities are here converted into toxicological units, applying the WHO’s Toxic Equivalency Factors for PCDD/Fs, as established in 2005 or 2022 (van den Berg et al. 2006; DeVito et al. 2024). It appears that the quantity of PCDD/Fs derived from measurements in Lausanne (425 gTEQ_WHO-2022_ or 371 gTEQ_WHO-2005_) is of the same order of magnitude as the modeled levels (1,283–1,698 gTEQ_WHO-2022_ or 1,115–1,419 gTEQ_WHO-2005_). A significant overestimation of the quantity derived by the model was expected, primarily due to the assumption in the validation analysis that all emitted PCDD/Fs have settled on the ground and remained within the boundaries of Lausanne.

A comprehensive description of the three validation methods, along with their corresponding results, can be found in the Supplementary Information [Validation Analysis]. Together, the three validation approaches tend to support the legitimacy of the proposed methodology.

### Sensitivity Analysis

Sensitivity analysis is run for scenario n°1 (1958–1982) and n°2 (1982–2005), considering the two cases of WS removal efficiency. In all scenarios, the top contributors to the variance in modeled PCDD/F emission quantity are Cl content (43–47%), temperature (27–30%), and waste flow rate (13–14%). The efficiencies of ESP and WS follow as additional contributors (3–8%). As expected, both waste-specific parameters and operational parameters are key factors in model uncertainty. It should be noted that contribution factors represent relative influences within the defined parameter space and should be interpreted as indicative.(Table 3).

**Table 3:** Top-5 input parameters contributing to the variance in modeled emission quantity and the contribution factors (in parentheses)

Scenario n°1	Scenario n°2	
	*η*_WS_ = 0%	*η*_WS_ = 40%
*f*_Cl_ (0.47)	*f*_Cl_ (0.46)	*f*_Cl_ (0.43)
*T* (0.30)	*T* (0.29)	*T* (0.27)
*m*_waste_ (0.14)	*m*_waste_ (0.14)	*m*_waste_ (0.13)
*η*_ESP_ (0.07)	*η*_ESP_ (0.07)	*η*_WS_ (0.08)
*f*_metal_ (0.02)	*η*_WS_ (0.03)	*η*_ESP_ (0.06)

## Limitations of the Approach

Overall, the proposed emission profile and quantity model provide a tool for estimating the magnitude and time evolution of PCDD/F emissions at the stack of an MSWI of interest. In the Vallon MSWI case, the model is, however, not without limitations due to data unavailability and simplifications. Literature data used for examining the effects of ESP and WS on PCDD/F emissions and congener distribution are selected based on the similarity to the configuration of the studied incinerator, but the difference in operating conditions necessitates adjustments. The corrections made to the available literature data are limited to accounting for temperature effects on phase partitioning of PCDD/Fs, while the temperature dependence of other parameters, such as de novo synthesis rate and the effect of other factors, including residence times, dust concentration, and ESP electric field strength, is not considered. Additionally, the validation analysis of the model is simplified to considering only the atmospheric deposition and half-lives in soil of the PCDD/F congeners. Half-life data are extracted from studies on paddy soil and sludge-amended soil, as these are the only available literature data; however, their applicability to the studied site is constrained by the potentially different soil properties, including pH, redox condition, and texture. Due to the overall uncertainty level inherent in various parameters arising from both the modeling and validation processes, the proposed methodology should undergo more robust validations in the future.

## Conclusion

A two-step model based on congener profile and emission quantity of PCDD/Fs at an MSWI is proposed in this study. The validation case study yielded a good agreement between measurements and modeling results for the Vallon MSWI in Switzerland.

As a future work, the model can be advanced by including additional processes and factors. For example, methods can be developed to model the influence of different APCD systems on PCDD/F emissions, taking into account the operational parameters of these systems. This would facilitate a case-specific understanding of how APCD characteristics influence PCDD/F emissions and allow for their integration into the model framework. As the model complexity increases, the importance of establishing an inventory of PCDD/F monitoring data becomes evident. The database should comprise incineration systems with diverse configurations, waste compositions, and operating conditions. This comprehensive database would enable various applications, such as identifying congener distribution patterns for typical waste compositions and estimating the magnitude of emission quantities associated with different APCD systems.

To facilitate application of this model to incinerators with limited site-specific historical data, it is recommended to prioritize local and national archival reports and literature to inform model inputs and assumptions before consulting international data. When using non-local data, similarity to the target incinerator should be evaluated based on comparable technology, capacity, and air pollution control measures for operational parameters, and on consumption patterns and waste management practices for waste-related inputs. Sensitivity analysis can help identify the most influential parameters and guide the emphasis of data collection efforts.

Overall, the developed model provides a useful analysis tool on the PCDD/F emission history at MSWIs and can assist in the evaluation of human exposure and health effects.

## Supplementary Information

Below is the link to the electronic supplementary material.

Supplementary file1 (DOCX 1077 KB)

## Data Availability

The proposed models are implemented in Python and made publicly available along with example input data in CSV format. Both the code and data can be accessed on Zenodo with https://doi.org/10.5281/zenodo.16634639, which archives the GitHub repository at https://github.com/Zhang-XC/dioxin-models.git. The code is released under the MIT License.

## Declarations

### Generative AI and AI-Assisted Technologies in the Writing Process

During the writing process, the authors used *DeepL* and *ChatGPT-4.0* to enhance the linguistic aspects and the academic formulation of certain paragraphs. After using these tools, the authors reviewed the content carefully and edited it as needed. The authors take full responsibility for the content of the published article.
