# Supplementary material for: Assessment of Past Dioxin Emissions from Waste Incineration Plants Based on Archive Studies and Process Modeling: A New Methodological Tool
Source: Arch Environ Contam Toxicol. 2025 Sep 8;89(3):252–65. doi: 10.1007/s00244-025-01150-9 (PMC12568895; doi:10.1007/s00244-025-01150-9)
Supplement: Supplementary file 1 — Supplementary file1 (DOCX 1077 KB) [file 244_2025_1150_MOESM1_ESM.docx]

Supplementary Information

Assessment of past dioxin emissions from waste incineration plants based on archive studies and process modeling: a new methodological tool

Xiaocheng Zhang^1^, Alexis de Aragao^1^, Fabien Moll-François^2,3^, Aurélie Berthet^3^, Florian Breider^1*^

^1^ Ecole Polytechnique Fédérale de Lausanne (EPFL), School of Architecture, Civil and Environmental Engineering, 1015 Lausanne, Switzerland

^2^ Ecole Polytechnique Fédérale de Lausanne (EPFL), College of Humanities, 1015 Lausanne, Switzerland

^3^ Center for Primary Care and Public Health (Unisanté), Department of Occupational and Environmental Health (DSTE), University of Lausanne, 1066 Epalinges-Lausanne, Switzerland

Xiaocheng Zhang and Alexis de Aragao are equal contributors to this work and designated as co-first authors

***Corresponding author: Florian Breider, phone: +41 21 693 08 69, florian.breider@epfl.ch**

# SUPPLEMENTARY INFORMATION – WHO Toxic Equivalency Factors of 17 PCDD/Fs

**Table S1** Toxicologically relevant PCDD/F congeners and their WHO-05 and WHO-22 toxic equivalency factors (van den Berg et al. 2006; DeVito et al. 2024)

| PCDD/F toxic congener | TEF WHO-05 | TEF WHO-22 |
| --- | --- | --- |
| 2,3,7,8-TCDD | 1 | 1 |
| 1,2,3,7,8-PeCDD | 1 | 0.4 |
| 1,2,3,4,7,8-HxCDD | 0.1 | 0.09 |
| 1,2,3,6,7,8-HxCDD | 0.1 | 0.07 |
| 1,2,3,7,8,9-HxCDD | 0.1 | 0.05 |
| 1,2,3,4,6,7,8-HpCDD | 0.01 | 0.05 |
| OCDD | 0.0003 | 0.001 |
| 2,3,7,8-TCDF | 0.1 | 0.07 |
| 1,2,3,7,8-PeCDF | 0.03 | 0.01 |
| 2,3,4,7,8-PeCDF | 0.3 | 0.1 |
| 1,2,3,4,7,8-HxCDF | 0.1 | 0.3 |
| 1,2,3,6,7,8-HxCDF | 0.1 | 0.09 |
| 1,2,3,7,8,9-HxCDF | 0.1 | 0.2 |
| 2,3,4,6,7,8-HxCDF | 0.1 | 0.1 |
| 1,2,3,4,6,7,8-HpCDF | 0.01 | 0.02 |
| 1,2,3,4,7,8,9-HpCDF | 0.01 | 0.1 |
| OCDF | 0.0003 | 0.002 |

# SUPPLEMENTARY INFORMATION – Waste Element Composition

In the proposed methodology, the mass fraction of chlorine in the input waste is a critical parameter in estimating the congener profile in a MSWI furnace. It is also included in the emission quantity model along with the content of other elements, such as carbon, hydrogen, oxygen, copper, and iron. A method is presented here for estimating the waste element contents relying on categorical waste composition. The waste composition may typically be deduced from archive documents. This involves the examination of MSWI’s incinerated waste inventories, investigation campaigns conducted in the MSWI supply basin, or surveys on household waste conducted at regional and national levels.

As shown in Table S2, eleven categories of waste components are considered, and the typical element content in each category is drawn from the literature. The fraction of C, H, O, S, Fe, and Cu in the plastic, textile, wood, miscellaneous combustible, organics, paper, glass, metal, and miscellaneous non-combustible components are collected from Riber et al. (2009) and converted to wet basis using the overall moisture content of the waste. The correspondences between the 48 categories used in their study and 9 categories of interest are listed in Table S3, and element percentage in each category is taken as the average of element content in the corresponding detailed categories weighted by waste amount. The content of Cl in the aforementioned 9 categories are assigned according to Themelis (2010). Element contents of the unclassified and undetermined miscellaneous components are taken to be the average of miscellaneous combustible and miscellaneous non-combustible. For the medical waste category, the contents of Fe, Cu, and Cl are set to the average of the range proposed in Liu et al. (2018), and the fractions of C, H, O, and S are taken from Li and Jenq (1993).

**Table S2** Element content (wt%) in each waste category

| Category | C | H | O | S | Fe | Cu | Cl |
| --- | --- | --- | --- | --- | --- | --- | --- |
| Paper | 36.05 | 4.87 | 32.18 | 0.07 | 0.09 | 0.00 | 0.20 |
| Textile | 48.94 | 5.64 | 32.69 | 0.37 | 0.03 | 0.00 | 1.25 |
| Wood | 43.34 | 5.33 | 25.36 | 0.07 | 0.08 | 0.00 | 1.25 |
| Plastic | 67.24 | 9.43 | 8.20 | 0.05 | 0.09 | 0.01 | 2.50 |
| Glass | 0.00 | 0.00 | 0.00 | 0.03 | 0.14 | 0.00 | 0.06 |
| Organics | 14.82 | 2.03 | 9.11 | 0.07 | 0.01 | 0.00 | 0.30 |
| Metal | 2.02 | 0.36 | 0.71 | 0.02 | 29.15 | 0.11 | 0.06 |
| Medical waste | 33.54 | 5.11 | 14.96 | 0.03 | 0.07 | 0.13 | 1.55 |
| Misc. combustible | 30.34 | 4.27 | 16.09 | 0.11 | 0.61 | 0.03 | 1.25 |
| Misc. non-combustible | 2.56 | 0.41 | 1.97 | 0.08 | 1.44 | 0.92 | 0.06 |
| Misc. not classified and not determined | 23.68 | 3.35 | 12.71 | 0.10 | 0.81 | 0.24 | 0.66 |

**Table S3** Correspondences between 9 categories of interest in this study and the 48 categories proposed in Riber et al. (2009)

| Categories in this study | Categories in Riber et al. (2009) |
| --- | --- |
| Paper | Dirty paper, Dirty cardboard, Magazines, Advertisements, Books and phonebooks, Office paper, Newsprints, Other clean paper, Paper and carton containers, Cardboard, Milk cartons and alike, Carton with Al-foil |
| Textile | Textiles |
| Wood | Wood |
| Plastic | Plastic bottles, Hard plastic, Soft plastic, Non-recyclable plastic |
| Glass | Clear glass, Green glass, Brown glass, Other glass |
| Organics | Veg. food, Animal food, Yard waste, Animals etc. |
| Metal | Al containers, Al trays/foil, Metal like foil, Metal containers, Other metal |
| Misc. combustible | Kitchen tissues, Cotton stick etc., Rubber etc., Cigarette butts, Diapers and tampons, Other cotton etc., Shoes, Leather, Office articles, Vacuum cleaner bags, Other combustibles |
| Misc. non-combustible | Soil, Ash, Ceramics, Cat gravel, Batteries, Stones and gravel, Other non-combustibles |

To obtain the waste element content, we first calculate the fraction of waste in each of the 11 categories listed in Table S2. The mass fraction of each element in the waste can then be derived as the average across all categories, with each category’s contribution weighted by its respective waste mass fraction.

Once the chlorine content of the target MSWI has been estimated, it is necessary to determine its alignment with a critical threshold of 0.8–1.1% by weight of chlorine (Cl-wt%). This range marks a distinction between two types of PCDD/F stack profiles (Wang et al. 2003). Below this threshold, PCDD fraction tends to dominate, characterized by high ratios of 1,2,3,4,6,7,8-HpCDD and OCDD congeners. Above the threshold, PCDF fraction tends to prevail, which is reflected by high ratios of 2,3,4,7,8-PeCDF, 1,2,3,4,7,8-HxCDF, 1,2,3,6,7,8-HxCDF, 2,3,4,6,7,8-HxCDF, 1,2,3,4,6,7,8-HpCDF, and 1,2,3,4,7,8,9-HpCDF congeners. Likely, below 0.8–1.1 Cl-wt%, preferential chlorination of unsubstituted phenol produces chlorophenols, crucial PCDD precursors. Chlorophenols probably further impede the catalytic sites responsible for PCDF formation from carbon, instead of chlorinating dibenzofuran. Above the 0.8–1.1 Cl-wt% threshold, combustion quality likely declines, leading to an increase of incomplete combustion products (ICPs). If PCDD/Fs are generated from ICPs within the incinerator, the formation rates of PCDFs outstrip those of PCDDs. For empirical estimation, data from reference incinerators with similar chlorine content or stack congener distribution to the threshold is therefore suitable.

# SUPPLEMENTARY INFORMATION – APCD Efficiency Adjustment

If PCDD/F studies on relative profiles do not provide any APCD efficiency data in terms of congener mass-concentrations but present information on congener mass-fractions, the following equation can be used, although less accurate:

| $R_{i,j_{\mathrm{out}}}= R_{i,j_{\mathrm{in}}}\cdot\frac{\left( 1+ \varepsilon_{i,j} \right)}{\underset{correction to ensure 100\% sum on congener fractions}{\underbrace{\sum_{k}^{17} \left[ \left( 1+ \varepsilon_{k,j} \right)\cdot R_{k,j_{\mathrm{in}}} \right]}}}$ | (1) |
| --- | --- |

where $\varepsilon_{i,j}$ is the mass-fraction-based efficiency of APC chain or the device $j$on the congener $i$.

Occasionally, an APC chain or device within the MSWI under investigation may exhibit variations in its overall efficiency for removing pollutants in either the particulate or gaseous phases when compared to similar components in reference incinerators. Such disparities are typically attributed to differences in design or the effects of aging. To address the differences in the removal of gas-phase and particulate-phase material between a target MSWI and a reference incinerator, one proposed solution is to adjust the PCDD/F profile at the output of an APC chain or device for the target MSWI. However, this adjustment necessitates acquiring profile data throughout the upstream treatment process of interest with the ability to distinguish between particulate and gaseous phases:

| $R_{i,j_{\mathrm{out}, \mathrm{phase} X}}= \underset{correction factor}{\underbrace{\left( \frac{\varphi_{\mathrm{MSWI},j_{, \mathrm{phase} X}}}{\varphi_{\mathrm{REF},j_{, \mathrm{phase} X}}} \right)}}\cdot\frac{\left( 1 - \eta_{i,j_{\mathrm{in}, phase X}} \right) \cdot R_{i,j_{\mathrm{in}, phase X}}}{\sum_{k}^{17} \left[ \left( 1 - \eta_{k,j_{\mathrm{in}, phase X}} \right)\cdot R_{k,j_{\mathrm{in}, phase X}} \right]}$ | (2) |
| --- | --- |

where $\varphi_{MSWI,j_{, \mathrm{phase} X}}$ denotes the mass-concentration-based efficiency of the APC chain or device $j$on overall material in phase *X* (gaseous or particulate) as estimated for the target MSWI. Similarly, $\varphi_{REF,j_{,\mathrm{phase} X}}$represents this variable with respect to the reference incinerators.

# SUPPLEMENTARY INFORMATION – PCDD/F Formation Mechanism

1. *Homogeneous synthesis (500*–*800°C)*, which occurs within a temperature range of 500–800°C (Stanmore 2004). It starts with the generation of chlorophenoxy radicals from organics and chlorine, followed by the formation of PCDD/Fs through the dimerization of chlorophenoxy radicals (Weber and Hagenmaier 1999). While of somewhat subordinate importance, this process provides precursors for the heterogeneous pathways (Peng et al. 2020).
2. *Heterogeneous de novo synthesis (200–400°C)*, which initiates at lower temperature windows of 200–400°C, stemming from residual carbon, a product of incomplete combustion, under catalytic chlorination by copper and other transitional metals (Peng et al. 2020). De novo synthesis has long been reported to exhibit congener-specific and significantly temperature-dependent formation rates (Kilgroe 1996).
3. *Heterogeneous precursor synthesis (200–400°C)*, which also occurs within the temperature window of 200–400°C (Stanmore 2004). It requires chlorinated organics structurally similar to PCDD/Fs as precursors and active sites on fly ash for adsorption and catalytic reactions of the precursors (Peng et al. 2020). This pathway is less significant than de novo synthesis in MSWIs, where precursor concentrations are typically below 10 μg/Nm^3^ (Everaert and Baeyens 2002).

# SUPPLEMENTARY INFORMATION – Emission Quantity Model

This section provides a detailed description of the kinetic model introduced in section 2.2 of the main script.

The model describes the dependence of PCDD/F formation on chlorine and metal content in the waste by introducing half saturation constants into the rate equation. The increase in chlorine and metal content contributes to the formation of PCDD/Fs, but this effect saturates at high levels of chlorine and metal content. The ratio of actual oxygen mass flow rate to the stoichiometrically needed oxygen supply is included in both reactions to account for the effect of oxygen content on PCDD/F synthesis and destruction. The waste’s oxygen demand is dependent on the waste element composition, estimated using the method in Supplementary Information [Waste Element Composition]. The temperature dependence of rate constants is described by the Arrhenius equation:

| $k_{i}=k_{0i}\exp\left( \frac{E}{RT} \right)$ | (3) |
| --- | --- |

where $k_{i}$ is the congener-specific rate constant, $k_{0i}$ is the pre-exponential factor, $E$ is the activation energy and is assumed constant for all congeners for simplicity.

The total amount of produced $\left[ PCDD/Fs \right]_{i}$ in the furnace is dependent on the mass of incinerated waste, and is given by:

| $\left[ PCDD/Fs \right]_{i,\mathrm{furnace}}=\left[ PCDD/Fs \right]_{i}\cdot m_{\mathrm{waste}}$ | (4) |
| --- | --- |

After having computed the generated amount of the specific congener within the target MSWI, the total generated PCDD/F amount is derived by factoring in the specific contribution of the individual congener to the total concentrations. This specific contribution is determined based on the pre-established relative profile after furnace:

| $\left[ PCDD/Fs \right]_{\mathrm{furnace}}=\left[ PCDD/Fs \right]_{i,\mathrm{furnace}}/n_{i}$ | (5) |
| --- | --- |

where $n_{i}$ is the share of congener $i$ in the initial relative profile of PCDD/Fs, after the furnace, and $\left[ PCDD/Fs \right]_{\mathrm{furnace}}$ is the total generated amount of the seventeen PCDD/Fs in the furnace.

The quantity of PCDD/Fs generated in the furnace can be further altered by APCDs in the post-combustion zone. To determine the effect of APCDs, it is advised to extract data from MSWIs with the same APCD configuration, comparable PCDD/F concentration in inlet flue gas, and similar operating conditions (see section 2.1 in the main script). The operational duration of the reference and studied MSWI should also preferably be similar. In cases where they do not align, adjustments for the aging of the devices should be performed to correct the data (see Supplementary Information [APCDs Efficiency Adjustment]).

Given the inlet and outlet concentrations of PCDD/Fs in the reference APCD system, the removal efficiency is derived as:

| $\eta=1-\frac{\left[ PCDD/Fs \right]_{\mathrm{out}}}{\left[ PCDD/Fs \right]_{\mathrm{in}}}$ | (6) |
| --- | --- |

Note that the removal efficiency can be negative in the case of PCDD/F formation in a suitable temperature window (see section 2.1.3 in the main script). The PCDD/F concentration at the outlet of the studied APCD can then be calculated from the inlet concentration and the reference removal efficiency.

Regarding the operational parameters specific to the Vallon MSWI, a furnace temperature of 950°C and a residence time of 2 seconds are assumed (Voelgyi 1985). The waste feed rate for each year is calculated from the annual incinerated waste amount, assuming an annual operating time of 8,050 hours. The combustion air flow rate is taken as the average of 8 measurements of the flue gas flow rate between 1967 and 1983, being 53,425 m^3^/h (Bulletins du Conseil communal 1980, 1984; Airmes AG 2021). The weight fraction of O_2_ in the combustion air is assumed to be 0.23. The weight fractions of C, H, O, S, Fe, Cu, and Cl are estimated for years 1960, 1969, 1982, 1990, and 2001, corresponding to the years for which waste composition records are available, and the values for each year are obtained by linear interpolation. In addition, it is assumed that the two parallel furnaces were operating under the same conditions.

# SUPPLEMENTARY INFORMATION – Sensitivity Analysis

A random sampling method is applied to understand the contribution of each input parameter to the uncertainty in the output, namely the annual PCDD/F stack emission at the Vallon MSWI. This method is adapted from MacLeod et al. (2002) and Luo and Yang (2007).

Latin Hypercube Sampling is used for generating the random samples of parameter combinations. The range of variation for each parameter is divided into equal-sized subintervals, and one value is drawn from each interval based on the probability density in the interval. The parameters are allowed to vary simultaneously to evaluate the distribution of model outputs over the whole parameter space. A sample size of 50,000 is chosen such that the mean and variance of the outputs are invariant over different sets of random inputs. The contribution of the $i^{\mathrm{th}}$ parameter to the variance in the output (denotes as $\sigma_{{O, I}_{i}}$) is described by:

| $\sigma_{{O, I}_{i}}^{2}=k_{i}\sigma_{O}^{2}$ | (7) |
| --- | --- |

where $k_{i}$ is the contribution factor of the $i^{\mathrm{th}}$ input, $\sigma_{O}$ is the variance in the output obtained from stochastic simulations. To compute the contribution factors $k_{i}$, the input and output values are transformed into ranks, from which the Spearman rank correlation coefficient is calculated:

| $\rho_{i}=\frac{1-6\sum_{i=1}^{N} d_{j}^{2}}{N^{3}-N}$ | (8) |
| --- | --- |

where $\rho_{i}$ is the rank correlation coefficient of the $i^{\mathrm{th}}$ parameter, $d_{j}$ is the rank difference between the $j^{\mathrm{th}}$ observation for the inputs and outputs, $N$ is the sample size. $\rho$ indicates the degree of monotonicity between the input and output. For $N>100$, Student’s $t$ provides an accurate test of the significance of $\rho_{i}$ (Zar 1972):

| $t=\frac{\rho_{i}}{\sqrt{(1-\rho_{i}^{2})/(N-2)}}$ | (9) |
| --- | --- |

The contribution factors $k_{i}$ are then calculated from $\rho_{i}$ as:

| $k_{i}=\frac{\rho_{i}^{2}}{\sum_{i} \rho_{i}^{2}}$ | (10) |
| --- | --- |

Table S4 lists all input parameters for the emission quantity model that are drawn from truncated normal distributions. For the parameters with series of measurements and estimates, the mean ($\mu$) and standard deviation ($\sigma$) are taken from the available data, while the lower and upper bounds are determined as two standard deviations away from the mean. For temperature and the removal efficiencies of ESP and WS, $\mu$ is taken as the input value of the emission quantity model, and $\sigma$ is chosen such that the possibly large variance of the parameter is respected. The lower and upper bounds of temperature distribution are drawn from the literature (Bunsan et al. 2013). Two separate ranges are proposed for the two cases of WS removal efficiency (0% and 40%). Additionally, oxygen content in the combustion air ($f_{\mathrm{oxygen}}$) and the fraction of OCDF in total PCDD/Fs at the furnace ($f_{\mathrm{OCDF}}$) are kept constant, and two intermediate parameters, residence time and oxygen ratio, are calculated from other independent variables (Table S5).

Simulations are run for the two scenarios: scenario n°1 (1958–1982) with the ESP as the only APCD, and scenario n°2 (1982–2005) with both the ESP and the WS, considering two cases of WS removal efficiency. The obtained rank correlation coefficients are summarized in Table S6. Parameters including air flow rate and the weight fractions of C, H, O, and S show no significant effect ($P>0.05$) on the variance in modeled emission quantity.

**Table S4** Input parameters used in the emission quantity model with assigned probability distributions

| Input | Definition | Parameters | | | |
| --- | --- | --- | --- | --- | --- |
|  |  | *Μ* | *σ* | min | Max |
| *T* | Temperature (°C) | 950 | 95 | 750 | 1100 |
| *m*_waste_ | Waste flow rate (kg/s) | 1.72 | 0.24 | 1.23 | 2.21 |
| *m*_air_ | Air flow rate (kg/s) | 18.93 | 5.20 | 8.52 | 29.34 |
| *η*_ESP_ | ESP removal efficiency | −1.61 | 0.24 | −2.20 | −1.00 |
| *η*_WS_ | WS removal efficiency | 0.00 | 0.06 | −0.20 | 0.20 |
|  |  | 0.40 | 0.06 | 0.20 | 0.60 |
| *f*_C_ | C content (wt/wt) | 0.248 | 0.021 | 0.206 | 0.290 |
| *f*_H_ | H content (wt/wt) | 0.034 | 0.003 | 0.028 | 0.040 |
| *f*_O_ | O content (wt/wt) | 0.159 | 0.014 | 0.132 | 0.186 |
| *f*_S_ | S content (wt/wt) | 8.0×10^−4^ | 3.1×10^−5^ | 7.3×10^−4^ | 8.6×10^−4^ |
| *f*_Cl_ | Cl content (wt/wt) | 0.005 | 0.001 | 0.002 | 0.008 |
| *f*_metal_ | Metal content (wt/wt) | 0.016 | 0.006 | 0.005 | 0.028 |

**Table S5** Input parameters used in the emission quantity model with fixed or calculated values

| Input | Definition | Value |
| --- | --- | --- |
| *f*_oxygen_ | Oxygen content in combustion air | 0.23 |
| *f*_OCDF_ | OCDF fraction in PCDD/Fs (wt/wt) | 0.207 |
| *t* | Residence time (s) | $\mu\left( m_{\mathrm{air}} \right)\cdot\mu\left( t \right){/m}_{\mathrm{air}}$ |
| *λ*_oxygen_ | Oxygen ratio ((kg/s)/(kg/s)) | $\lambda_{\mathrm{oxygen}}=\frac{m_{\mathrm{air}}\cdot f_{\mathrm{oxygen}}\cdot100+m_{\mathrm{waste}}\cdot\%O}{m_{\mathrm{waste}}\left( \frac{\%C}{12}+\frac{\%H}{4}+\frac{\%S}{32} \right)\cdot32}$ |

**Table S6** Spearman rank correlation coefficient ($\rho$) for each input parameter in scenarios n°1 and n°2 and under two cases of WS removal efficiency ($\eta_{\mathrm{WS}}$)

| Input | Scenario n°1 | Scenario n°2 | |
| --- | --- | --- | --- |
|  |  | *η*_WS_ = 0% | *η*_WS_ = 40% |
| *T* | −0.53* | −0.52* | −0.51* |
| *m*_waste_ | 0.36* | 0.36* | 0.35* |
| *m*_air_ | 4.84×10^−3^ | 4.67×10^−3^ | 4.39×10^−3^ |
| *η*_ESP_ | −0.25* | −0.25* | −0.24* |
| *η*_WS_ |  | −0.16* | −0.27* |
| *f*_C_ | 6.65×10^−3^ | 6.69×10^−3^ | 6.50×10^−3^ |
| *f*_H_ | 6.13×10^−3^ | 5.99×10^−3^ | 5.88×10^−3^ |
| *f*_O_ | −2.30×10^−3^ | −2.14×10^−3^ | −1.94×10^−3^ |
| *f*_S_ | 2.37×10^−3^ | 5.35×10^−4^ | −7.18×10^−4^ |
| *f*_Cl_ | 0.67* | 0.66* | 0.64* |
| *f*_metal_ | 0.14* | 0.14* | 0.14* |

* *ρ* is significant at the 0.05 level

# SUPPLEMENTARY INFORMATION – Vallon History

The project of a MSWI plant in Lausanne was discussed as early as 1947 and the decision to build the incinerator was definitively taken in 1954. The choice for the construction of a waste incinerator was dictated by the lack of capacity of Lausanne’s landfills. It was then foreseen that the Vallon landfill, consisting of a filling of the Flon Valley, would be saturated by the end of 1956 (Bulletins du Conseil communal 1954).

The design of the incinerator itself began in 1954. It was decided to build the MSWI plant in the Vallon sector, located in Lausanne urban area, which benefited from the vicinity of the Vallon landfill for the deposit of future slag, the proximity of the thermal plant of Pierre-de-Plan for the production of heat, as well as a good overall spatial situation in the waste supply area (Bulletins du Conseil communal 1954).

The plant was sized considering both the characteristics of the supply area and the projection of the waste quantities in the future (Bulletins du Conseil communal 1954). Consequently, the Vallon MSWI was designed for a mass incineration capacity of about 83,000 tons, considering an average lower heating value of 5,000 KJ/kg. In addition, around 29,000 tons of waste were expected to be incinerated during the first year of operation. The resulting incinerator consisted of a 900 m^3^ waste pit, with two furnaces of 5.5 Gcal/h heat capacity each. The combustion gas produced in the furnace was then to be used to feed boilers for heat recovery, with a thermal efficiency of 51%. The generated steam was to be sent to the Pierre-de-Plan plant, located 400 m away (Voelgyi 1985). Finally, the combustion gases were then to be roughly filtered and released into the atmosphere through an 80 m high stack (Bulletins du Conseil communal 1954).

The incinerator was commissioned on October 6, 1958 (Syfrig 1958). The following years, the Vallon MSWI plant experienced a strong development phase. The number of supplying communes increased from 5 in 1958 to 55 in 1977 (Municipalité de Lausanne 1958, 1977). Over the same period, the annual amount of incinerated urban waste went from 200 to 324 kg/inhabitant (Municipalité de Lausanne 1984). The quantity of waste incinerated at the MSWI therefore expanded sharply. As a result, the incinerator was already operating 3×8 hours in 1964 (Bulletins du Conseil communal 1964). The Vallon MSWI further reached its saturation point in 1968 (Bulletins du Conseil communal 1970, 1990). That year, a record amount of 60,763 tons of waste was processed at Vallon (Municipalité de Lausanne 1968). Simultaneously, the waste lower heating value increased linearly from 5,000 KJ/kg in 1958 to 11,715 KJ/kg in 1988 (Bulletins du Conseil communal 1988; Voelgyi 1985). The rise of the lower heating value is mostly explained by changes in the composition of the waste over time (e.g. continuous growth of the plastic fraction). This led to a more and more reduced incineration capacity at the Vallon MSWI. The amount of waste fed into the incinerator therefore exceeded the incineration capacity from 1969 (Voelgyi 1985). For example, the theoretical capacity was only 39,727 tons in 1982, while 47,437 tons were actually incinerated that year (for more information, please refer to Supplementary Information [Vallon History of Waste Properties and Combustion Quality]). These deplorable operating conditions induced a poor quality of combustion at the Vallon incinerator, i.e. incomplete combustion. As a glaring figure, the mass of incineration residues (including slag and ash) exceeded 50% of the total mass incinerated in 1972, 1973 and 1981 (for more information, please refer to Supplementary Information [Vallon History of Waste Properties and Combustion Quality]).

From 1980 onwards, the quantity of waste incinerated at the Vallon slightly decreased. Three factors explain this. First, the number of neighboring communes supplying the MSWI fell, with only 3 remaining in 1985 (Municipalité de Lausanne 1985). Second, sorting policies were expanding at that time (Municipalité de Lausanne 1984). Third, the arrival of a compactor at the Vallon allowed for the economical evacuation of surplus waste to other sites, such as Monthey, from 1986 (Municipalité de Lausanne 1987). In parallel, the capacity of the incinerator increased in the 80’s, due to both renovation works undertaken in 1983 and a slowdown in the increase of the calorific value of waste (please refer to Supplementary Information [Vallon History of Waste Properties and Combustion Quality]). Thus, under the combined action of the reduction in the quantity of waste incinerated and the increase in incineration capacity, the Vallon MSWI was no longer overloaded in the 1990s. Until its closure in 2005, the Vallon plant nevertheless remained saturated and the combustion conditions, although improved, remained poor, especially with the presence of too many unburnt residues and high carbon monoxide emissions (as indicated in documents collected from the archives of the former municipal incinerator and currently held by Tridel S.A. in Lausanne).

During its lifetime (1958–2005), the plant incinerated a wide variety of solid waste. The vast majority was household waste. Industrial waste, waste from public services and administrations, WWTP bar screens, bulky household waste, special waste, confidential or infectious waste and hospital waste represented a smaller proportion. Details on the time-evolution of the type and quantity of waste, its average lower heating value, the proportion of incineration residues and the incinerator capacity are given in the Supplementary Information [Vallon History of Waste Properties and Combustion Quality].

From an environmental point of view, the Lausanne incinerator was seen as progress at the time of its conception. Until then, the waste was simply spread on the fields or deposited in sites, called “ruclons” (Syfrig 1958). However, from the very first years of operation, environmental adverse impacts were noted. The Vallon plant emitted into the air a lot of small residues of waste, called “bruchons”, product of the incomplete combustion (Bulletins du Conseil communal 1960). In an attempt to solve this issue, a series of optimization of the ash treatment occurred between 1960 and 1966, with, in particular, the installation of rotary screens in 1961 and a third electroprecipitator in 1966 (Bulletins du Conseil communal 1971, 1978). These measures did not solve the problem satisfactorily (Bulletins du Conseil communal 1975; Rollier et al. 1983). In addition to the “bruchons” pollution, the Vallon incinerator turned out to be an important source of heavy metal contamination for the surrounding soil (Quinche 1983, 1984). It also may have contributed to the acidification of the rain locally (Voelgyi 1985). These various environmental impacts were finally addressed by the installation of wet scrubbers in 1982, which showed tremendous positive effects on “bruchons” and other emitted pollutants, including dust, heavy metals and chlorinated acids. However, mercury emissions remained above the regulatory threshold at Vallon plant despite the installation of wet scrubbers (Bulletins du Conseil communal 1984). Nevertheless, from the end of the 1980s onwards, the dilapidated state of the plant was frequently highlighted. As early as 1988, the City of Lausanne presented the incineration furnaces as being the oldest in the world still in operation (Bulletins du Conseil communal 1988). Moreover, the Vallon plant did not fully meet the new 1992 legal requirements of the Federal Ordinance on Air Pollution Control (referred to as OAPC in English, LRV in German, oPair in French), as reported in the press in the late 1990s (Isler 1999). In order to satisfy the legal obligations stipulated in the OAPC, the Vallon MSWI had therefore to be brought into compliance no later than 2002 (Bulletins du Conseil communal 2000). This was never carried out and the Vallon incinerator continued to operate until 29 December 2005 and the commissioning of a brand new MSWI: Tridel (Municipalité de Lausanne 2005). Fifteen years after its closure, the Vallon plant is once again in the spotlight as the source of a widespread dioxin soil pollution in Lausanne (Etat de Vaud 2021).

# SUPPLEMENTARY INFORMATION – Vallon Technical Aspects


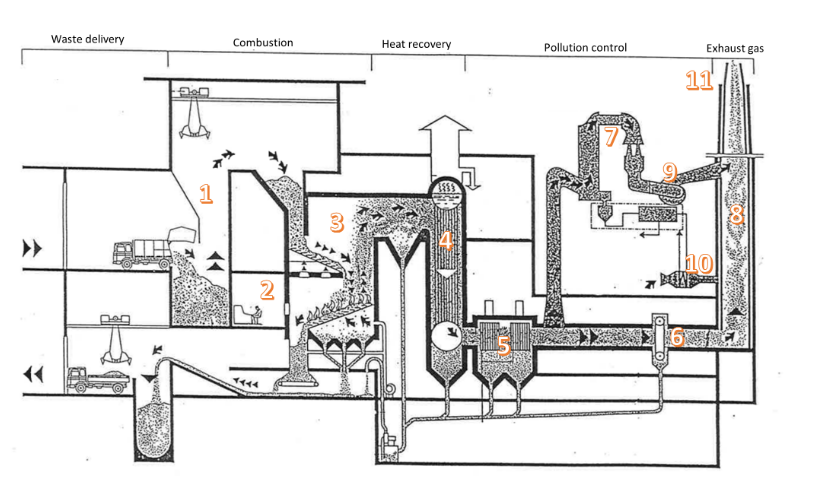


**Fig. S1** Schematic of the Vallon MSWI plant, illustration from Rollier et al. (1983), caption modified

The different steps of waste incineration at the Vallon plant are described hereafter, with the numbers in brackets referring to the elements depicted in the Fig. S1.

(1) First, the solid waste was dumped into a 900 m^3^ capacity pit (Voelgyi 1985).

(2) Under the supervision of the operator located in the control room, the waste was lifted by a crane, and dropped into a hopper (Voelgyi 1985).

(3) Through the hopper, the waste entered the combustion chamber. The incineration process took place in two “Von Roll” furnaces commissioned in 1958, which could operate in parallel or in single-line. The average annual operating hours per furnace are estimated to be about 8,050 since at least 1968 (Jeanneret 1969; Voelgyi 1985; Municipalité de Lausanne 1983–1989). In the furnaces, the waste first slipped over a pre-drying grate of 5.7 m^2^ and then onto the main incineration grate of 8.0 m^2^. Further combustion of difficult-to-burn materials was enabled in the gasification shaft. To prevent bottom ash to agglomerate, exhaust steam was injected from below. The temperature in the incineration chamber reached 950±50°C (Voelgyi 1985).

(4) The hot flue gases then exited the furnace and entered the afterburner area. There, they were cooled to 280–300°C by exchanging heat with a boiler having a total exchange surface of 616 m^2^ (Rollier et al. 1983; Voelgyi 1985).

(5) The gases then underwent cleaning treatments. As early as 1958, two electrostatic precipitators were in operation. In 1966, a third precipitator was implemented. The electrostatic precipitators could work all together, in combination or individually by opening and closing valves (Rollier et al. 1983).

(6) Extraction mats were added to the filtration system in May 1960, followed by the implementation of temporary dust vacuum cleaners in June 1961. They were placed after the electrostatic precipitators, before the stack discharge. These devices were replaced by rotary screens and new types of temporary vacuum cleaners in October 1961. In July 1962, the definitive vacuum cleaners were finally installed. When the third electrostatic precipitator was commissioned in 1966, it was also equipped with rotary screens and vacuum cleaners (Bulletins du Conseil communal 1978).

(7) In 1982, a new era in pollution control began at the Vallon MSWI, with the addition to the existing system of a wet gas scrubber, developed by the LAB company. The wet scrubber was installed after the electrostatic precipitators. It was composed of two units that could process flue gas in parallel or in a single line. Each unit was composed of three hydraulic circuits (Voelgyi 1985):

1. Gas cooling: the incoming gases had a temperature of about 250°C (Rollier et al. 1983). Water was neutralized with caustic soda and sprayed on the combustion gases. Coarse dusts (>50 μm) were largely removed and part of the hydrochloric acid was eliminated.
2. Absorption tower: water, also neutralized with caustic soda, was sprayed on the combustion gases. The PH level remained between 5 and 5.5. This allowed filtration of suspended dusts, neutralization of sulfur dioxide and the reduction of hydrochloric and hydrofluoric acids concentrations.
3. Electrodynamic Venturi: an adiabatic expansion of the combustion gases took place in a Venturi, causing the condensation of the supersaturated gases on the surfaces of fine dust (condensation core). The dust became moist and negatively charged by ionization. The dust was then attracted by positively charged water droplets and could be discharged together.

It should be noted that the Lausanne wet scrubber was the first developed by LAB to be used to treat flue gases from a MSWI (Bulletins du Conseil communal 1980).

(8) The fumes from both lines were next released into the atmosphere through a common 80 m high chimney. The exhaust gas temperature was around 250 to 300°C before the wet scrubber implementation (Rollier et al. 1983). After the wet gas scrubber introduction, gases were discharged through the stack at a much lower temperature (60–65°C). For safety reasons, the gases could still bypass the gas scrubber system and be directly discharged through the stack (Voelgyi 1985). According to measurements made between 1967 and 1983, the volumetric flow rate of the flue gases ranged from 47,600 to 81,500 m^3^ dry gas/h, when the two lines were running (Airmes AG 2021).

To compensate for the thermal drop of the exhaust gas after the installation of the gas scrubber, some elements were added in 1982 (Voelgyi 1985):

(9) First, a fan was used to accelerate the gases towards the stack after each gas scrubber line.

(10) Secondly, the space between the concrete and the brickwork of the chimney was traversed by a flow of pressurized hot air of temperature between 100 and 120°C. The latter was heated thanks to an air/scrubber water heat exchanger (HEX) and another air/vapor HEX.

(11) Finally, an ejection cone was built at the top of the stack to increase the ejection velocity to 16 m/s.

The archival records provide key information on the air pollution control measures, but data on the disposal of bottom ash and wet scrubber sludge is absent. The developed modeling framework does not attempt to address all discharge pathways, and focuses on giving an estimate to stack emission based on limited technical information.

# SUPPLEMENTARY INFORMATION – Vallon History of Waste Properties and Combustion Quality

Table S7 presents the evolution of waste characteristics and combustion quality at the Vallon MSWI between 1959 and 2005. It includes, for each year, the lower heating value of the waste, the mass capacity of the incinerator, the amount of waste incinerated, the amount of waste evacuated, the types of waste processed, and the fraction of incineration residues (including slag, ash, and WS sludge).

Unless otherwise stated, the data used in Table S7 is taken from the Annual Management Reports of the Lausanne municipality from 1959 to 2005 (Municipalité de Lausanne 1959–2005).

Regarding the lower heating value and the incinerator capacity, data for the period 1959–1983 is taken from the article by Voelgyi (1985), who was the head of Lausanne’s sanitation department from 1979 to 1994. This is completed by the figures provided by the Bulletins of the City Council of Lausanne for the years 1988, 1990 and 2000 (Bulletins du Conseil communal 1988, 1990, 2000).

Concerning the quantity of waste incinerated at the Vallon MSWI, only processed quantities are reported by the Lausanne Municipality until 1982, and the quantities incinerated are not indicated. A reasonable approximation is made here that the quantity of waste incinerated is equivalent to the quantity processed up to 1982. This may nevertheless lead to a slight overestimation of the annual quantity of waste incinerated. It is important to additionally note that the total amount of waste treated/incinerated does not always correspond perfectly to the sum of materials treated/incinerated as reported by the raw data (positive or negative deviation depending on the case). Nevertheless, where a discrepancy does exist, it is negligible in proportion (2.5% maximum, most of the time < 0.5%).

Regarding the quantity of waste evacuated, the Lausanne Municipality reports evacuations only from 1982 (Municipalité de Lausanne 1983). However, it cannot be excluded that evacuations were carried out at the Vallon the preceding years. It should be noted that between 1982 and 1985, evacuations were conducted because of the unavailability of the MSWI due to renovation works (Municipalité de Lausanne 1985). From 1986, waste evacuations gained in magnitude thanks to the arrival of a compactor, allowing the systematic economical evacuation of surplus waste from the Vallon to other sites (Municipalité de Lausanne 1987).

For the household waste fraction, the household waste amount reported by the Lausanne Municipality as coming from communes other than Lausanne is added to the household waste amount reported by the Lausanne Municipality as coming from the commune of Lausanne (for the period 1959–1972 and 1982–2005) or collected in Lausanne (for the period 1973–1981). In connection with major renovation works in 1978, 6,370 tons of household waste collected in Lausanne were directly sent to the landfills of Châtel-St-Denis and Penthaz to compensate for the unavailability of the furnaces at Vallon (2,334 cumulative hours). This diverted quantity is therefore subtracted from the quantity collected in Lausanne, to obtain the quantity actually delivered to the Vallon that year.

With regard to the tire waste fraction, the quantity of tires provided by the Lausanne Municipality for the year 1975 obviously contains an error and is probably given in units of kilograms and not in tons. The conversion to tons is therefore made. In addition to the data provided in the Annual Management Reports for the years 1975 to 1978, documents available in the cantonal archives provide figures for the years 1979 and 1980.

Finally, the calculation of the fraction of incineration residues is done by considering the ratio of incineration residues quantity (including here slag, ash, and WS sludge) to the total amount of waste incinerated. According to archive documents from the former Vallon MSWI, it should be noted that the WS wastewater was sent directly to the WWTP until 1987 and no WS sludge was therefore managed as incineration residues between 1982 and 1987. Between 1987 and 1996, WS residual sludge was mixed with the slag. Finally, the residual sludge from the WS was no longer mixed with the slag from 1996 onwards but instead dehydrated and disposed separately in a landfill (Municipalité de Lausanne 1996). This likely explains the apparent decrease in incineration residues fraction after 1996 (i.e. drying-related sludge weight loss). Moreover, from 1997 onwards, ash from the electrostatic precipitators was no longer hydraulically discharged and mixed with slag, but was evacuated using a pneumatic system, then stabilized and disposed of separately in a landfill (Bulletins du Conseil communal 1952, 1984; Municipality of Lausanne 1997). Once again, this may explain the slight drop in the proportion of incineration residues after 1997 (i.e. less wet residues).

According to Fig. S2, the average lower heating value of the waste continuously increased during the period of operation of the Vallon MSWI (from about 5,000 to 11,000 KJ/kg between 1959 and 1983), notably due to the spread of plastics. As a consequence, the capacity of the incinerator decreased almost proportionally (from about 83,000 tons in 1959 to 40,000 tons in 1982). In parallel, the amount of waste to be incinerated rose sharply at first (from about 29,000 tons in 1959 to about 61,000 tons in 1968) and then remained generally stable until 1979. These competing trends led to a saturation of the MSWI already in 1968, and then to an overload in the following years, i.e. the amount of waste incinerated exceeded the capacity of the incinerator. The quality of the combustion was therefore deplorable at the Vallon during this early phase (with the incredible figure of more than 50% of incineration residues produced in 1972, 1973 and 1981).

However, from the 1980s until the closure of the Vallon MSWI in 2005, the quality of combustion gradually improved (e.g. only 25% of incineration residues was recorded in 2001). This amelioration is mostly explained by three factors. First, major repairs were undertaken to counter the severe state of degradation of the Vallon plant. Thus, the gasifier of one of the two furnaces which exploded on 27 April 1982 as well as the furnaces that threatened to collapse, were renovated and even improved in 1983 (Bulletins du Conseil communal 1983). This allowed the incinerator capacity to go back to about 47,500 t/year, otherwise it would have fallen below 36,000 t/year (Bulletins du Conseil communal 1988). The other gasifier was additionally replaced in 1990 (Municipalité de Lausanne 1990). The furnaces also underwent further revisions in 1999 and 2001 (Municipalité de Lausanne 1999, 2001). Second, from the mid-80s onwards, the growth in the lower heating value of waste slowed down considerably to a plateau, reducing the negative contribution to the incinerator mass capacity. Third, the amount of waste incinerated at the Vallon MSWI decreased slightly from the early 1980s. This was made possible by a drop in the number of communes supplying the Vallon (Municipalité de Lausanne 1958–2005), the emergence of sorting policies (Municipalité de Lausanne 1984), as well as the arrival of a compactor at the Vallon in 1986 allowing the economical evacuation of surplus waste to other sites (Municipalité de Lausanne 1987). By 1990, although the furnaces were still saturated, they were therefore no longer overloaded. The combined effects of an increase in incineration capacity and a decrease in the quantity of waste incinerated yielded a positive effect on combustion quality (i.e. smaller incineration residues fraction). Nevertheless, on the basis of documents collected in the archives, the combustion conditions remained unsatisfactory until the closure of the Vallon MSWI in 2005, with high carbon monoxide emissions and too much unburnt material in the slag, non-compliant with the Swiss Federal Ordinance on the Avoidance and the Disposal of Waste (referred to as ADWO in English, TVA in German, OTD in French).


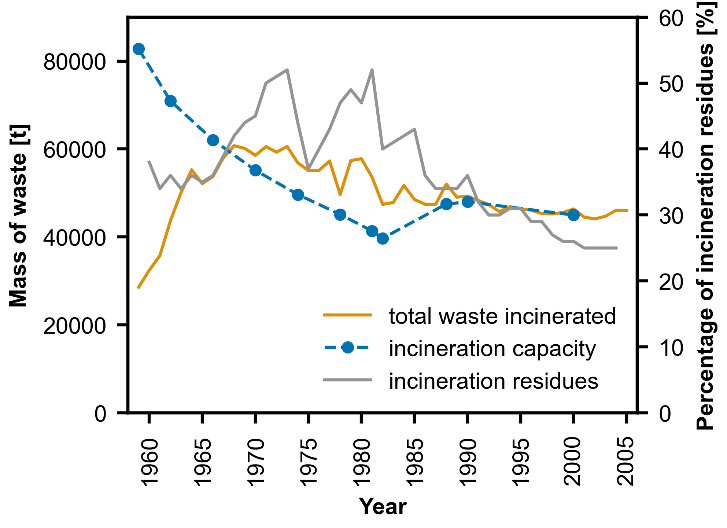


**Fig. S2** Time evolution (1959–2005) in incineration capacity, total mass of waste incinerated and fraction of incineration residues produced (secondary axis) at the Vallon MSWI plant

**Table S7** Time evolution (1959–2005) of lower heating value of waste, incinerator capacity, mass of waste incinerated, mass of waste evacuated, types of waste processed and fraction of incineration residues at Vallon MSWI plant

| Year | Lower heating value [kJ/kg] | Waste incineration capacity [t] | Incinerated waste [t] | Evacuated waste [t] | Household waste [t] | Private industrial waste [t] | Commune services [t] | Vidy bar screens [t] | Cantonal administration services [t] | Tires [t] | Residues (slag & ash & WS sludge) |
| --- | --- | --- | --- | --- | --- | --- | --- | --- | --- | --- | --- |
| 1959 | 5021 | 82764 | 28590 |  | 23950 | 4640 |  |  |  |  |  |
| 1960 |  |  | 32436 |  | 27000 | 4000 | 1000 |  |  |  | 38% |
| 1961 |  |  | 35763 |  | 29715 | 5098 | 950 |  |  |  | 34% |
| 1962 | 5858 | 70940 | 43725 |  | 36316 | 6876 | 533 |  |  |  | 36% |
| 1963 |  |  | 50127 |  | 41559 | 8193 | 375 |  |  |  | 34% |
| 1964 |  |  | 55290 |  | 44617 | 10091 | 582 |  |  |  | 36% |
| 1965 |  |  | 52135 |  | 42113 | 9214 | 808 |  |  |  | 35% |
| 1966 | 6694 | 62073 | 53745 |  | 42502 | 10486 | 707 |  |  |  | 36% |
| 1967 |  |  | 58053 |  | 45468 | 11222 | 372 | 991 |  |  | 39% |
| 1968 |  |  | 60763 |  | 46870 | 12753 | 138 | 1002 |  |  | 42% |
| 1969 |  |  | 60158 |  | 47362 | 11577 | 150 | 1069 |  |  | 44% |
| 1970 | 7531 | 55176 | 58564 |  | 44635 | 12531 | 103 | 1295 |  |  | 45% |
| 1971 |  |  | 60559 |  | 45348 | 13294 | 87 | 1840 |  |  | 50% |
| 1972 |  |  | 59300 |  | 45836 | 11583 | 51 | 1830 |  |  | 51% |
| 1973 |  |  | 60592 |  | 47187 | 11929 | 74 | 1879 |  |  | 52% |
| 1974 | 8368 | 49658 | 56868 |  | 44380 | 10519 | 203 | 1765 |  |  | 44% |
| 1975 |  |  | 55116 |  | 44215 | 9027 | 350 | 1520 |  | 144 | 37% |
| 1976 |  |  | 55139 |  | 43918 | 8515 | 486 | 1406 |  | 809 | 40% |
| 1977 |  |  | 57296 |  | 46522 | 8102 | 278 | 1294 |  | 1098 | 43% |
| 1978 | 9205 | 45144 | 49655 |  | 40321 | 7855 | 215 | 835 |  | 341 | 47% |
| 1979 |  |  | 57375 |  | 48277 | 7686 | 229 | 1377 |  | 302 | 49% |
| 1980 |  |  | 57809 |  | 47815 | 7185 | 347 | 1341 |  | 227 | 47% |
| 1981 | 10042 | 41382 | 53617 |  | 46117 | 6266 | 385 | 848 |  |  | 52% |
| 1982 | 10460 | 39727 | 47437 | 2889 | 42078 | 6516 | 464 |  |  |  | 40% |
| 1983 | 10710 |  | 47853 | 2910 | 42863 | 5192 | 964 |  | 1744 |  | 41% |
| 1984 |  |  | 51720 | 669 | 44394 | 5309 | 891 |  | 1795 |  | 42% |
| 1985 |  |  | 48535 | 2140 | 43730 | 4131 | 1102 |  | 1691 |  | 43% |
| 1986 |  |  | 47457 | 5007 | 44989 | 4458 | 1175 |  | 1842 |  | 36% |
| 1987 |  |  | 47448 | 7560 | 47392 | 4487 | 1257 |  | 1872 |  | 34% |
| 1988 | 11715 | 47500 | 52028 | 4142 | 48172 | 4875 | 1196 |  | 1927 |  | 34% |
| Year | Lower heating value [kJ/kg] | Waste incineration capacity [t] | Incinerated waste [t] | Evacuated waste [t] | Household waste [t] | Industrial waste [t] | Household bulky waste [t] | Confidential and infectious waste [t] | Hospital waste [t] |  | Residues (slag & ash & WS sludge) |
| 1989 |  |  | 49058 | 6759 | 46882 | 6317 | 2351 | 240 | 37 |  | 34% |
| 1990 | 10878 | 48000 | 49305 | 4595 | 46260 | 4412 | 1905 | 346 | 997 |  | 36% |
| 1991 |  |  | 48307 | 4953 | 45131 | 3887 | 2042 | 448 | 1752 |  | 32% |
| 1992 |  |  | 47392 | 4338 | 43466 | 3902 | 2209 | 443 | 1710 |  | 30% |
| 1993 |  |  | 45784 | 3859 | 41755 | 3610 | 2245 | 569 | 1464 |  | 30% |
| 1994 |  |  | 46900 | 2151 | 40344 | 4428 | 2698 | 617 | 964 |  | 31% |
| Year | Lower heating value [kJ/kg] | Waste incineration capacity [t] | Incinerated waste [t] | Evacuated waste [t] | Household waste [t] | Industrial waste [t] | Household bulky waste [t] | Confidential waste [t] | Hospital and infectious waste [t] | Special waste [t] | Residues (slag & ash & WS sludge) |
| 1995 |  |  | 46363 | 1984 | 39515 | 4491 | 2796 | 544 | 1000 | 1 | 31% |
| 1996 |  |  | 46131 | 2606 | 44063 | 22 | 2996 | 593 | 1040 | 23 | 29% |
| 1997 |  |  | 45322 | 2477 | 42884 | 67 | 2949 | 820 | 1024 | 55 | 29% |
| 1998 |  |  | 45259 | 3058 | 43162 | 82 | 3261 | 701 | 1045 | 66 | 27% |
| 1999 |  |  | 45583 | 4724 | 44927 | 127 | 3045 | 709 | 1073 | 66 | 26% |
| 2000 |  | 45000 | 46379 | 5610 | 45904 | 80 | 3901 | 730 | 1262 | 62 | 26% |
| 2001 |  |  | 44515 | 8810 | 47103 | 101 | 4033 | 666 | 1252 | 70 | 25% |
| 2002 |  |  | 44126 | 10111 | 47934 | 101 | 4091 | 752 | 1265 | 94 | 25% |
| 2003 |  |  | 44715 | 9753 | 47937 | 83 | 4394 | 666 | 1278 | 110 | 25% |
| 2004 |  |  | 46070 | 9567 | 48541 | 83 | 4809 | 805 | 1356 | 42 | 25% |
| 2005 |  |  | 46041 |  |  |  |  |  |  |  |  |

# SUPPLEMENTARY INFORMATION – Vallon Congener Profile Estimation

A method for extrapolating the PCDD/F congener profile at the outlet of the ESP in the Vallon, based on measurements performed by Chang et al. (2004) at an incinerator in Taiwan is here proposed. It first consists in correcting the effect of the temperature at the ESP entrance (234°C in Taiwan versus 290°C at Vallon) on the gas/particulate phase distributions for the Vallon. Then, the outlet ESP profile at Vallon is derived thanks to the phase-specific removal efficiencies reported by Chang et al. (2004). These efficiencies are previously adjusted based on assumptions regarding the ageing of the ESP at Vallon.

Table S8 displays the data from the Taiwanese MSWI. In column a), the ratios of the 17 congeners to the total PCDD/F concentration before the ESP are indicated, as well as the gas/particle phase distribution. Column b) refers to the situation after the ESP. Finally, column c) lists the absolute (concentration-based) removal efficiencies of the ESP on the congeners.

The proposed method for extrapolating the congener profile at Vallon is detailed in successive steps hereafter:

1. A regression between the congener vapor pressures and the congener shares in gaseous phase is computed based on empirical relationships at atmospheric pressure, for a temperature of 234°C (ESP inlet temperature at the Taiwanese MSWI). It is assumed here that the vapor pressures of the different congeners are an important explanatory factor for the congener distribution in gaseous or particulate phase. This is in line with statements by Chang et al. (2004). The gas phase shares are directly computed from the data measured by Chang et al. (2004) at the inlet of the ESP, whereas the vapor pressures at 234°C are estimated using the empirical relationship proposed by Paasivirta et al. (1999). The latter relates the vapor pressure $p$ (in Pascals) of each congener to the surrounding temperature $T$ (in Kelvins) through the expression:

| $\log_{10}\left( p \right)= A- \frac{B}{T}$ | (11) |
| --- | --- |

For each PCDD/F congener, the parameters $A$ and $B$ are extracted from Table 10 of the paper by Paasivirta et al. (1999), for supercooled liquid. Regarding the congener 2,3,7,8-TeCDD, a more reliable experiment-based empirical relationship is proposed by Schroy et al. (1985) and is thus incorporated in the regression. This empirical expression is valid for a wide range of temperature applications (10°C to 305°C) and relates the 2,3,7,8-TeCDD vapor pressure $p$ (in Pascals) to the surrounding temperature $T$ (in Kelvins) as follows:

| $\ln\left( p \right)= 34.57083- \frac{14903.438}{T}$ | (12) |
| --- | --- |

It is important to note the uncertainty in such empirical estimates. Indeed, the method of Paasivirta et al. (1999) gives a value of 67.2 Pa for the 2,3,7,8-TeCDD congener, while that of Schroy et al. (1985) yields 178.4 Pa at 234°C. The vapor pressures estimated for a temperature of 234°C and atmospheric pressure are given in Table S9.

**Table S8** PCDD/F profile before and after the ESP, along with ESP (concentration-based) removal efficiencies, categorized by gaseous (G.), particulate (P.), and total (G. + P.) phases, for a MSWI investigated by Chang et al. (2004)

| Congener | a) Before ESP  (% PCDD/F conc.) | | | b) After ESP  (% PCDD/F conc.) | | | c) ESP removal efficiency  (% conc.) | | |
| --- | --- | --- | --- | --- | --- | --- | --- | --- | --- |
|  | G. | P. | G. + P. | G. | P. | G. + P. | G. | P. | G. + P. |
| 2,3,7,8-TeCDD | 0.1 | 0.1 | 0.2 | 0.1 | 0.0 | 0.1 | −173 | 68 | −74 |
| 1,2,3,7,8-PeCDD | 0.3 | 0.4 | 0.7 | 0.8 | 0.2 | 1.0 | −479 | 1 | −212 |
| 1,2,3,4,7,8-HxCDD | 0.3 | 0.5 | 0.8 | 0.9 | 0.3 | 1.2 | −631 | −23 | −247 |
| 1,2,3,6,7,8-HxCDD | 0.5 | 1.0 | 1.5 | 2.2 | 0.7 | 2.9 | −857 | −57 | −340 |
| 1,2,3,7,8,9-HxCDD | 0.4 | 0.6 | 1.0 | 1.3 | 0.4 | 1.6 | −610 | −30 | −259 |
| 1,2,3,4,6,7,8-HpCDD | 2.8 | 5.6 | 8.4 | 10.1 | 3.4 | 13.5 | −719 | −37 | −264 |
| OCDD | 8.3 | 17.7 | 26.0 | 17.4 | 6.8 | 24.2 | −380 | 13 | −112 |
| 2,3,7,8-TeCDF | 0.7 | 0.4 | 1.0 | 1.1 | 0.1 | 1.2 | −265 | 34 | −156 |
| 1,2,3,7,8-PeCDF | 0.8 | 0.9 | 1.8 | 1.7 | 0.3 | 2.0 | −363 | 19 | −160 |
| 2,3,4,7,8-PeCDF | 1.8 | 1.8 | 3.6 | 4.1 | 0.9 | 5.0 | −429 | −9 | −217 |
| 1,2,3,4,7,8-HxCDF | 1.0 | 1.6 | 2.7 | 2.6 | 0.7 | 3.3 | −484 | 0 | −186 |
| 1,2,3,6,7,8-HxCDF | 1.4 | 2.2 | 3.6 | 3.5 | 1.0 | 4.4 | −470 | −2 | −184 |
| 1,2,3,7,8,9-HxCDF | 0.1 | 0.2 | 0.3 | 0.3 | 0.1 | 0.4 | −403 | −14 | −196 |
| 2,3,4,6,7,8-HxCDF | 3.5 | 4.0 | 7.5 | 6.5 | 1.9 | 8.3 | −320 | −6 | −154 |
| 1,2,3,4,6,7,8-HpCDF | 6.8 | 10.9 | 17.7 | 11.4 | 3.7 | 15.1 | −283 | 22 | −94 |
| 1,2,3,4,7,8,9-HpCDF | 1.1 | 1.6 | 2.7 | 2.0 | 0.7 | 2.7 | −312 | 2 | −127 |
| OCDF | 7.6 | 13.1 | 20.7 | 9.4 | 3.7 | 13.1 | −180 | 36 | −44 |
| Σ PCDDs | 13 | 26 | 39 | 33 | 12 | 44 | −489 | −2 | −162 |
| Σ PCDFs | 25 | 37 | 61 | 42 | 13 | 56 | −290 | 19 | −106 |
| Σ PCDDs + PCDFs | 37 | 63 | 100 | 75 | 25 | 100 | −357 | 10 | −127 |

**Table S9** Vapor pressures of PCDD/F congeners at 234°C, based on empirical relationships from Paasivirta et al. (1999) or Schroy et al. (1985) in blue

| Congener | Coefficients | | $\log_{10}p\left( 234^{\circ}C \right)$  [log(Pascal)] | $p(234^{\circ}C)$ [Pascal] |
| --- | --- | --- | --- | --- |
|  | A | B |  |  |
| 2,3,7,8-TeCDD | 15.01 | 6472 | 2.25 | 178.4 |
| 1,2,3,7,8-PeCDD | 8.38 | 3321 | 1.83 | 67.9 |
| 1,2,3,4,7,8-HxCDD | 8.37 | 3769 | 0.94 | 8.7 |
| 1,2,3,6,7,8-HxCDD | 8.47 | 3751 | 1.07 | 11.9 |
| 1,2,3,7,8,9-HxCDD | 8.07 | 3699 | 0.78 | 6.0 |
| 1,2,3,4,6,7,8-HpCDD | 7.95 | 3844 | 0.37 | 2.4 |
| OCDD | 8.32 | 4221 | 0.00 | 1.0 |
| 2,3,7,8-TeCDF | 8.66 | 3513 | 1.73 | 54.1 |
| 1,2,3,7,8-PeCDF | 8.23 | 3529 | 1.27 | 18.7 |
| 2,3,4,7,8-PeCDF | 7.90 | 3462 | 1.07 | 11.9 |
| 1,2,3,4,7,8-HxCDF | 7.81 | 3564 | 0.78 | 6.1 |
| 1,2,3,6,7,8-HxCDF | 7.91 | 3954 | 0.11 | 1.3 |
| 1,2,3,7,8,9-HxCDF | 7.90 | 3662 | 0.68 | 4.8 |
| 2,3,4,6,7,8-HxCDF | 8.00 | 3651 | 0.80 | 6.3 |
| 1,2,3,4,6,7,8-HpCDF | 7.45 | 3486 | 0.58 | 3.8 |
| 1,2,3,4,7,8,9-HpCDF | 7.42 | 3731 | 0.06 | 1.2 |
| OCDF | 7.77 | 4068 | -0.25 | 0.6 |

Finally, the following regression relating a congener vapor pressure to its gaseous phase share is found:

| $\alpha_{i,\mathrm{gas}}=0.0404 \cdot\ln\left( p_{i} \right)+0.35$ | (13) |
| --- | --- |

where $\alpha_{i,\mathrm{gas}}$ is the share in the gaseous phase of the $i^{\mathrm{th}}$ congener and $p_{i}$ is the vapor pressure of the $i^{\mathrm{th}}$ congener in Pascal.

The Pearson correlation coefficient of the regression amounts to *R* = 0.74, which confirms the strong correlation between the congener vapor pressures and the congener shares in gaseous phase.

1. The vapor pressures of the congeners at 290°C are then estimated applying the same empirical relationships as in point 1. The regression previously obtained is further used to derive the gas/particulate phase shares for each congener at 290°C, which corresponds to the average ESP inlet temperature at the Vallon MSWI. The distribution of congeners in the gaseous phase at the inlet of the Vallon ESP is then directly estimated by multiplying the derived gas phase shares for Vallon by the total-phase fractions observed at the inlet of the Taiwanese ESP. The congener distribution in the particulate phase is then simply derived by subtracting the distribution in the gas phase from the congener profile in the total phase.
2. The congener distributions after the ESP at Vallon are next estimated based on the phase-specific removal efficiencies computed from Chang et al. (2004) data. These are previously adjusted to take into account the ageing of the ESP in Lausanne. A 1.25 times less effective ESP for filtering particle-bound PCDD/Fs is thus considered at Vallon (it commissioned in 1958 versus 1994 for Taiwan). This 1.25 figure corresponds to the ratio between the upper and lower range of ESP removal efficiencies on particles (99%/80%) as indicated by the literature (White 1957). The assumption of a low efficiency at Vallon is justified by numerous evidence of a serious malfunction of the ESPs there, as reported by archival documents. In fact, between 1960 and 1962, the ESPs had to be constantly improved to overcome their poor efficiency. A third ESP was further implemented in 1966 (Bulletins du Conseil communal 1978). It aimed to reduce the velocity of flue gas in the filters from 1.67 m/s to 0.86 m/s in order to compensate for the still insufficient dust removal of the existing system (Bulletins du Conseil communal 1964). However, the results of these successive upgrades on dust emissions were limited (Bulletins du Conseil communal 1975). In the early 1970s, ESP failures were reported (Bulletins du Conseil communal 1971). In 1988, unpredictable breakdowns due to wear and tear, fatigue of the whole electro-mechanical part and corrosion of the static part were mentioned (Bulletins du Conseil communal 1971). In 1997, the third ESP had to be renovated (Municipalité de Lausanne 1997). Finally, in 2000, work was planned (but never carried out) to bring the Vallon MSWI into compliance with the OAPC. At the time, the two first ESPs installed in 1958 were described as insufficiently reliable and efficient (Bulletins du Conseil communal 2000). The figure of a low dust removal efficiency of 80% is also expressly proposed by Voelgyi (1985) for the Vallon case.

Table 2 of the main script displays the estimates obtained for the Vallon MSWI. In column a), the ratios of the 17 congeners to the total PCDD/F concentration before the ESP are indicated, as well as the gas/particle phase distribution. Column b) refers to the situation after the ESP. Finally, the column c) lists the absolute (concentration-based) removal efficiencies of the ESP on the congeners. It can be noticed that the higher ESP inlet temperature and the lower ESP efficiency at Vallon appear to have little effect on the ESP outlet congener profile. The difference in the profile found after adjustment is most likely within the uncertainties of the profile used. Therefore, the applied correction does not appear, in hindsight, to be necessary. It should be noted that the influence of de novo synthesis rate (*T*-dependent), as well as suspend particle concentrations, has not been included in this adjustment process.

The removal efficiencies of the WS on congener fractions as measured by Takaoka et al. (2003) and Chang et al. (2004) are presented in Table S10, columns a) and b). The average WS removal efficiencies on the total congener fractions (gas + particulate phases) are calculated in column c). These averages are normalized in column d) to ensure that the sum of the derived PCDD/F fractions after the WS is equal to 100%. It can be observed that the uncertainties on the fraction removal efficiencies of the WS are very large. Further research is necessary to confirm the reliability of these preliminary results on post-WS congener distribution.

Finally, Table S11 summarizes PCDD/F profile before the ESP, after the ESP and after the WS, as estimated for the Vallon MSWI.

**Table S10** WS (fraction-based) removal efficiencies, categorized by gaseous (G.), particulate (P.), and total (G. + P.) phases, as estimated for the Vallon MSWI

| Congener | a) Chang et al. | | | b) Takaoka et al. | | c) Average | d) Corrected average |
| --- | --- | --- | --- | --- | --- | --- | --- |
|  |  |  |  | MSWI-A | MSWI-B |  |  |
|  | G. % | P. % | G. + P. % | G. + P. % | G. + P. % | G. + P. % | G. + P. % |
| 2,3,7,8-TeCDD | 42 | −300 | 15 | 95 | 50 | 54 | 74 |
| 1,2,3,7,8-PeCDD | 71 | −76 | 45 | 83 | 33 | 54 | 74 |
| 1,2,3,4,7,8-HxCDD | 66 | −138 | 20 | 51 | 6 | 26 | 42 |
| 1,2,3,6,7,8-HxCDD | 68 | −163 | 14 | 54 | −6 | 21 | 37 |
| 1,2,3,7,8,9-HxCDD | 64 | −183 | 10 | 46 | 0 | 19 | 34 |
| 1,2,3,4,6,7,8-HpCDD | 56 | −268 | −25 | 3 | −60 | −27 | −18 |
| OCDD | 68 | −267 | −26 | −17 | −58 | −34 | −25 |
| 2,3,7,8-TeCDF | 77 | −64 | 64 | 96 | 49 | 69 | 92 |
| 1,2,3,7,8-PeCDF | 72 | −67 | 50 | 89 | 41 | 60 | 81 |
| 2,3,4,7,8-PeCDF | 73 | −41 | 53 | 82 | 33 | 56 | 77 |
| 1,2,3,4,7,8-HxCDF | 69 | −97 | 33 | 51 | 30 | 38 | 56 |
| 1,2,3,6,7,8-HxCDF | 71 | −94 | 34 | 52 | 25 | 37 | 55 |
| 1,2,3,7,8,9-HxCDF | 55 | −200 | 3 | 44 | −4 | 14 | 29 |
| 2,3,4,6,7,8-HxCDF | 69 | −78 | 37 | 17 | −6 | 16 | 31 |
| 1,2,3,4,6,7,8-HpCDF | 62 | −161 | 7 | −40 | 7 | −9 | 3 |
| 1,2,3,4,7,8,9-HpCDF | 53 | −177 | −6 | −46 | −39 | −30 | −21 |
| OCDF | 59 | −220 | −20 | −125 | −35 | −60 | −54 |
| Σ PCDDs | 64 | −253 | −19 | −1 | −38 | −19 | −17 |
| Σ PCDFs | 65 | −147 | 15 | 1 | 19 | 11 | 15 |
| Σ PCDDs + PCDFs | 65 | −197 | 0 | 0 | 0 | 0 | 0 |

**Table S11** PCDD/F profile before the ESP, after the ESP and after the WS for the total (G. + P.) phase, as estimated for the Vallon MSWI

| Congener | Before ESP | Scenario n°1  After ESP | Scenario n°2  After ESP and WS |
| --- | --- | --- | --- |
|  |  |  |  |
|  | G. + P. % | G. + P. % | G. + P. % |
| 2,3,7,8-TeCDD | 0.2 | 0.1 | 0.0 |
| 1,2,3,7,8-PeCDD | 0.7 | 1.0 | 0.3 |
| 1,2,3,4,7,8-HxCDD | 0.8 | 1.3 | 0.7 |
| 1,2,3,6,7,8-HxCDD | 1.5 | 3.3 | 2.1 |
| 1,2,3,7,8,9-HxCDD | 1.0 | 1.7 | 1.1 |
| 1,2,3,4,6,7,8-HpCDD | 8.4 | 14.6 | 17.2 |
| OCDD | 26.0 | 25.5 | 31.8 |
| 2,3,7,8-TeCDF | 1.0 | 1.0 | 0.1 |
| 1,2,3,7,8-PeCDF | 1.8 | 1.9 | 0.4 |
| 2,3,4,7,8-PeCDF | 3.6 | 4.5 | 1.0 |
| 1,2,3,4,7,8-HxCDF | 2.7 | 3.4 | 1.5 |
| 1,2,3,6,7,8-HxCDF | 3.6 | 4.2 | 1.9 |
| 1,2,3,7,8,9-HxCDF | 0.3 | 0.4 | 0.2 |
| 2,3,4,6,7,8-HxCDF | 7.5 | 7.5 | 5.1 |
| 1,2,3,4,6,7,8-HpCDF | 17.7 | 15.1 | 14.6 |
| 1,2,3,4,7,8,9-HpCDF | 2.7 | 2.4 | 2.9 |
| OCDF | 20.7 | 12.3 | 19.0 |
| Σ PCDDs | 39 | 47 | 53 |
| Σ PCDFs | 61 | 53 | 47 |
| Σ PCDDs + PCDFs | 100 | 100 | 100 |

# SUPPLEMENTARY INFORMATION – Vallon Wet Scrubber Mass Balance

For the effect of the WS on emission quantity, the following mass balance scheme is considered:

| $Input - Adsorption + Desorption + Synthesis = Output (gas) + Output (scrubbing solution)$ | (14) |
| --- | --- |

The inlet flue gas temperature at the Vallon WS was measured to be 250–269°C (Bulletins du Conseil communal 1984), which supports heterogeneous synthesis inside the WS. The Adsorption on the WS surface, packing materials, and scrubbing solution serves as a source for later Desorption. During the lifetime of the WS, there are periods when Adsorption dominates and periods when Desorption dominates, which can lead to great fluctuations in the outlet concentration. Here, it is assumed that Adsorption equals Desorption in each year of the WS operating period, and the amount remaining in the WS at the end of its operation is neglected. With these simplifications, it follows that the effect of the WS on PCDD/F emission is exclusively through the discharge from scrubbing solution and heterogeneous synthesis on fly ash.

# SUPPLEMENTARY INFORMATION – Validation Analysis

Three distinct methods are used to validate the proposed methodology, namely VA1, VA2 and VA3. They are here introduced, and rely on the specific Vallon pollution case:

1. VA1 is a validation method of the emission relative profile, portion of the model developed. It consists in comparing the congener distribution for the particulate phase at the Vallon ESP resulting from the model with the distribution measured at the Vallon in 1996. The latter was taken in the washwater of the ESP’ ash (and not in exhaust gas samples). This is the only time known to date that PCDD/Fs were analyzed at the Vallon plant (eOde 2022).

The congener profile estimated in the fly ash from the flue gas at the Vallon ESP (assumed to be the average between the pre-ESP and post-ESP particulate-phase profiles, see section 3.2 of the main script) is therefore compared to that measured in the washing water of the ESP ash in 1996. Note that caution should be taken when comparing the estimated profile in the fly ash with the profile found in the washing water of the ESP ash since the nature of the medium differs and, therefore, the underlying physicochemical processes affecting the distribution of congeners may diverge. In particular, the congener adsorbed on the fly ash have different sorption/desorption properties, as well as different dissolution capacities in the washing solution. Nevertheless, the comparison of the congener profile estimated in the fly ash (i.e. particulate PCCD/Fs) to that measured in the washing water solution seems to be an interesting tool to assess the validity of the empirical model that is used for the ESP description by both the scenario n°1 (1958–1982) and n°2 (1982–2005). It turns out that the correlation between the congener fractions estimated in the fly ash at the ESP and the fractions measured in the ESP washing water in 1996 is excellent with a correlation coefficient score of 0.98 (see Fig. S3). Regarding the standard deviation, it amounts to 2.6% on congener ratios and can be considered as good. These findings therefore tend to validate the part of the model on the relative congener profile estimation.


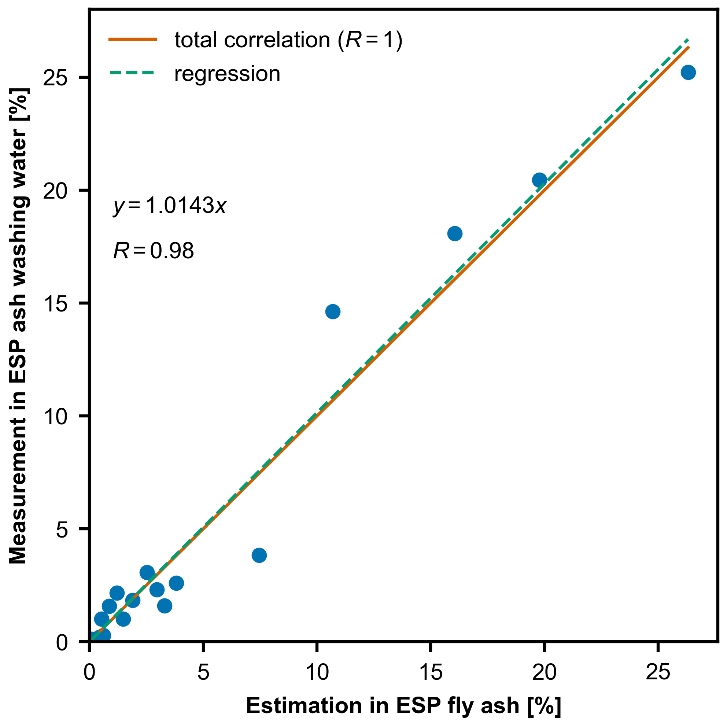


**Fig. S3** Fractions of PCDD/F congeners as estimated in the fly ash from flue gas at the ESP and as measured in the ESP washing solution in 1996 at Vallon. Regression line between the two data sets (green dashed line) with display of the regression equation and the Pearson’s coefficient

VA2 is a second, more sophisticated and holistic validation method. It first calculates the Vallon annual emission amounts of each congener from the congener profile estimated in section 3.2 of the main script, and the total annual PCDD/F emissions estimated in section 3.3 of the main script. It then integrates the results over time to 2022 by accounting for degradation of the congeners in soil based on their half-lives. A correction for soil sorption phenomena is also priory performed. Finally, the residual soil profile obtained is compared to a reference soil profile, calculated from the weighted average of soil measurements carried out in 2021–2022 in the Lausanne region.

As a first step, a notional amount of deposition to soil is calculated for each year and congener, based on the soil congener fractions and the estimated total amount of PCDD/Fs emitted from the Vallon MSWI for that year:

| $q_{i,j}= \alpha_{i, j, \mathrm{soil}}\cdot Q_{j}$ | (15) |
| --- | --- |

where $q_{i,j}$ represents the notional amount of the $i^{\mathrm{th}}$ congener deposited in soil during the $j^{\mathrm{th}}$ year, $\alpha_{i, j,\mathrm{soil}}$ is the estimated soil fraction of the $i^{\mathrm{th}}$ congener for the $j^{\mathrm{th}}$ year, and $Q_{j}$ is the estimated amount of PCDD/Fs (comprising the 17 relevant congeners) emitted by the MSWI during the $j^{\mathrm{th}}$ year.

It is important to recall that due to the large uncertainties on the wet scrubber efficiency (see section 3.3 of the main script), and the sensitivity of the model results to this parameter value (please refer to Supplementary Information [Sensitivity Analysis]), two scenarios for estimating PCDD/F emissions have been carried out: (1) with a WS efficiency of $\eta_{\mathrm{WS}}$= 0%, (2) with WS efficiency of $\eta_{\mathrm{WS}}$ = 40%. Therefore, two simulated time-integrated profiles will be here provided, each built on different datasets of the estimated annual amount of PCDD/Fs emitted by the MSWI.

In addition, the congener fractions in soil, denoted $\alpha_{i, j,\mathrm{soil}}$, are derived from the congener fractions in air, denoted $\alpha_{i, j,\mathrm{atm}}$, estimated in the scenarios n°1 (1958–1982) and n°2 (1982–2005) (see section 3.2 of the main script). The latter must be adjusted to consider the influence of deposition physico-chemistry on the congener profile. Although Wallenhorst (1996) observed marked similarity between congener patterns in air and soil deposition at three sampling sites, he reported that OCDD made an increasing contribution to the total PCDD/F concentration from 25% in air to 40% in soil. Therefore, the OCDD fraction in soil is boosted by a factor of 1.6 compared to those estimated for air. The other congener fractions are redistributed in consequence to match 100% sum on congener ratios (see Table S12). According to previous studies, it should be noted that environmental transport phenomena do not appear to affect significantly PCDD/F congener distribution (Wallenhorst 1996; Jones and Duarte-Davidson 1997).

**Table S12** Fractions of congeners in air, as estimated in scenarios n°1 and n°2 (see section 3.2 of the main script), and fractions of congeners in soil, adjusted according to Wallenhorst’s observations (Wallenhorst 1996)

| Congener | $\alpha_{i, j,\mathrm{atm}}$  sc. n°1 | $\alpha_{i, j,\mathrm{soil}}$  sc. n°1 | $\alpha_{i, j,\mathrm{atm}}$  sc. n°2 | $\alpha_{i, j,\mathrm{soil}}$  sc. n°2 |
| --- | --- | --- | --- | --- |
| 2,3,7,8-TeCDD | 0.1% | 0.1% | 0.0% | 0.0% |
| 1,2,3,7,8-PeCDD | 1.0% | 0.8% | 0.3% | 0.2% |
| 1,2,3,4,7,8-HxCDD | 1.3% | 1.0% | 0.7% | 0.5% |
| 1,2,3,6,7,8-HxCDD | 3.3% | 2.6% | 2.1% | 1.5% |
| 1,2,3,7,8,9-HxCDD | 1.7% | 1.3% | 1.1% | 0.8% |
| 1,2,3,4,6,7,8-HpCDD | 14.6% | 11.6% | 17.2% | 12.4% |
| OCDD | 25.5% | 40.7% | 31.8% | 50.9% |
| 2,3,7,8-TeCDF | 1.0% | 0.8% | 0.1% | 0.1% |
| 1,2,3,7,8-PeCDF | 1.9% | 1.5% | 0.4% | 0.3% |
| 2,3,4,7,8-PeCDF | 4.5% | 3.5% | 1.0% | 0.8% |
| 1,2,3,4,7,8-HxCDF | 3.4% | 2.7% | 1.5% | 1.1% |
| 1,2,3,6,7,8-HxCDF | 4.2% | 3.3% | 1.9% | 1.4% |
| 1,2,3,7,8,9-HxCDF | 0.4% | 0.3% | 0.2% | 0.2% |
| 2,3,4,6,7,8-HxCDF | 7.5% | 5.9% | 5.1% | 3.7% |
| 1,2,3,4,6,7,8-HpCDF | 15.1% | 12.0% | 14.6% | 10.5% |
| 1,2,3,4,7,8,9-HpCDF | 2.4% | 1.9% | 2.9% | 2.1% |
| OCDF | 12.3% | 9.8% | 19.0% | 13.7% |

Once the notional amounts annually deposited in the soil have been estimated for each congener, a time integration is applied to determine the remaining amount for each congener in 2022, by applying a first-order decay law:

| $q_{i,\mathrm{int},2022}= \sum_{j=1958}^{2005} q_{i,j}\cdot\exp\left( -\lambda_{i}\cdot\left( 2022-j \right) \right)$ | (16) |
| --- | --- |

where $q_{i,\mathrm{int}, 2022}$ is the notional residual amount of the $i^{\mathrm{th}}$ congener in the soil in 2022, and $\lambda_{i}$ denotes the decay constant of the $i^{\mathrm{th}}$ congener.

The decay constants $\lambda_{i}$ are derived from the half-lives of each congener:

| $\lambda_{i}= \frac{\ln(2)}{\tau_{1/2,i}}$ | (17) |
| --- | --- |

where $\tau_{1/2,i}$ is the half-life of the $i^{\mathrm{th}}$ congener.

To determine the first-order rate constants $\lambda_{i}$, a literature search is conducted on the half-lives of PCDD/Fs in soil, denoted $\tau_{1/2,i}$. Two papers studying PCDD/F degradation in a Japanese rice field soil and a sludge-amended soil are considered, by default, as the most transposable to the Lausanne soil context (McLachlan et al. 1996; Seike et al. 2007). Half-life values are directly extracted from Seike et al. (2007) whereas they must be calculated from the 1972 and 1985 field measurements provided by McLachlan et al. (1996). Half-lives of 16 of the 17 PCDD/F congeners are obtained from these two studies (see Table S13). That of 1,2,3,7,8,9-HxCDF is missing from both papers and is replaced by the average half-life of the HxCDFs. Finally, the two datasets are corrected to integrate the effect of temperature. The *Q*_10_ rule is applied, i.e. a 10°C increase in temperature results in a 2.2-fold increase in the decay constant (Sinkkonen and Paasivirta 2000). The soil temperature in Lausanne is approximated to be 10.7°C (which corresponds to the mean annual atmospheric temperature between 1959 and 2022 there). Finally, the half-lives from both datasets are averaged (see Table S13). These mean values are considered the reference half-lives in the rest of the analysis.

**Table S13** Half-lives in years as measured by Seike et al. (2007) and McLachlan et al. (1996) after temperature-adjustment applying the *Q*_10_ rule, and the average values used as reference half-lives. Half-life of the 1,2,3,7,8,9-HxCDF congener (blue row) is estimated by averaging HxCDF’s half-lives

| Congener | Seike et al. | McLachlan et al. | Average |
| --- | --- | --- | --- |
| 2,3,7,8-TeCDD | 6.4 | 11.6 | 9.0 |
| 1,2,3,7,8-PeCDD | 21.0 | 12.4 | 16.7 |
| 1,2,3,4,7,8-HxCDD | 17.4 | 10.2 | 13.8 |
| 1,2,3,6,7,8-HxCDD | 19.0 | 11.1 | 15.1 |
| 1,2,3,7,8,9-HxCDD | 13.6 | 12.6 | 13.1 |
| 1,2,3,4,6,7,8-HpCDD | 15.9 | 12.8 | 14.4 |
| OCDD | 14.8 | 11.7 | 13.3 |
| 2,3,7,8-TeCDF | 30.5 | 12.2 | 21.4 |
| 1,2,3,7,8-PeCDF | 15.1 | 19.4 | 17.3 |
| 2,3,4,7,8-PeCDF | 18.1 | 12.9 | 15.5 |
| 1,2,3,4,7,8-HxCDF | 25.4 | 14.2 | 19.8 |
| 1,2,3,6,7,8-HxCDF | 13.4 | 17.2 | 15.3 |
| 1,2,3,7,8,9-HxCDF | 21.7 | 16.6 | 19.2 |
| 2,3,4,6,7,8-HxCDF | 26.3 | 18.4 | 22.4 |
| 1,2,3,4,6,7,8-HpCDF | 11.8 | 14.5 | 13.2 |
| 1,2,3,4,7,8,9-HpCDF | 9.1 | 14.7 | 11.9 |
| OCDF | 13.1 | 13.0 | 13.1 |

The estimate of the soil congener profile in 2022, as derived from the model developed in this project, can then be trivially obtained from the estimated residual quantity of congeners in the soil in 2022:

| $\alpha_{i,\mathrm{int},2022}= \frac{q_{i,\mathrm{int},2022}}{\sum_{j=1}^{17} q_{j,\mathrm{int},2022}}$ | (18) |
| --- | --- |

where $\alpha_{i,\mathrm{int},2022}$ represents the fraction of the $i^{\mathrm{th}}$ congener in the estimated soil profile for the year 2022. The values of $\alpha_{i,\mathrm{int},2022}$ are given for the 17 congeners in Table S14.

Regarding the reference soil congener profile (whose fractions are denoted $\alpha_{i,\mathrm{meas},2021-22}$), it is calculated based on 124 soil measurements obtained from five surveys performed between March 2021 and June 2022 in Lausanne area and neighboring municipalities (Impact-Concept SA 2022). The first four investigations employed accelerated solvent extraction for sample analysis, while the last investigation used Soxhlet extraction. On the recommendation of cantonal directive, the analyses of PCDD/Fs in soils and excavation materials should be carried out using the Soxhlet extraction method (DGE 2021). To correct for the discrepancy introduced by soil extraction methods, measurements above the limit of quantification (LOQ) from the first four investigations are multiplied by a factor of 0.575, which is obtained from cross-analysis of 20 samples (Impact-Concept SA 2022). Additionally, measurements below the LOQ are assigned the value of 50% LOQ. The fraction $\alpha_{i,\mathrm{meas},2021-22}$ of the $i^{\mathrm{th}}$ congener is then calculated as the sum of its concentrations to the sum of all congeners in all sampling sites, which can be think of as a weighted average of the profiles. The resulting fraction values, denoted $\alpha_{i,\mathrm{meas},2021-22}$, are listed in Table S14.

**Table S14** Estimated soil profile based on temporal integration of 1958–2005 estimates to 2022 ($\alpha_{i,\mathrm{int},2022}$ fractions) and reference soil profile based on weighted average of soil profile measurements performed between 2021 and 2022 ($\alpha_{i,\mathrm{meas},2021-22}$ fractions)

| Congener | $\alpha_{i,\mathrm{int},2022}$  ($\eta_{\mathrm{WS}}$ = 0%) | $\alpha_{i,\mathrm{int},2022}$  ($\eta_{\mathrm{WS}}$ = 40%) | $\alpha_{i,\mathrm{meas},2021-22}$ |
| --- | --- | --- | --- |
| 2,3,7,8-TeCDD | 0.0% | 0.0% | 0.2% |
| 1,2,3,7,8-PeCDD | 0.4% | 0.5% | 1.7% |
| 1,2,3,4,7,8-HxCDD | 0.6% | 0.7% | 2.0% |
| 1,2,3,6,7,8-HxCDD | 2.0% | 2.1% | 2.4% |
| 1,2,3,7,8,9-HxCDD | 0.8% | 0.8% | 2.6% |
| 1,2,3,4,6,7,8-HpCDD | 12.7% | 12.6% | 18.4% |
| OCDD | 44.8% | 43.2% | 34.8% |
| 2,3,7,8-TeCDF | 0.5% | 0.6% | 0.6% |
| 1,2,3,7,8-PeCDF | 0.8% | 1.0% | 1.5% |
| 2,3,4,7,8-PeCDF | 1.7% | 2.0% | 1.8% |
| 1,2,3,4,7,8-HxCDF | 2.4% | 2.8% | 3.3% |
| 1,2,3,6,7,8-HxCDF | 2.1% | 2.3% | 3.3% |
| 1,2,3,7,8,9-HxCDF | 0.3% | 0.3% | 0.2% |
| 2,3,4,6,7,8-HxCDF | 7.9% | 8.7% | 3.1% |
| 1,2,3,4,6,7,8-HpCDF | 9.8% | 9.8% | 16.8% |
| 1,2,3,4,7,8,9-HpCDF | 1.6% | 1.5% | 1.2% |
| OCDF | 11.6% | 11.0% | 6.0% |

Finally, the soil profile estimated for the year 2022 (whose fractions are $\alpha_{i,\mathrm{int},2022}$) is compared with the reference soil profile for the years 2021–2022 (whose fractions are $\alpha_{i,\mathrm{meas},2021-22}$). Both data are shown under the form of congener-sorted bars in Fig. S4. Indicative uncertainties are also calculated and added to the estimated fractions. They are established by considering variations of −50% to +100% on the reference half-lives previously defined (see Table S13). The choice of a range of variation from −50% to +100% is arbitrary but legitimate considering the large divergence on the half-lives of PCDD/Fs proposed in the literature. To construct the uncertainty bars, one million simulations have been performed by randomly selecting half-lives within the predefined ranges of variation for each congener. Then, the 2.5% and 97.5% quantiles of the resulting fractions define the lower and upper limits of the error bar for each congener.

**
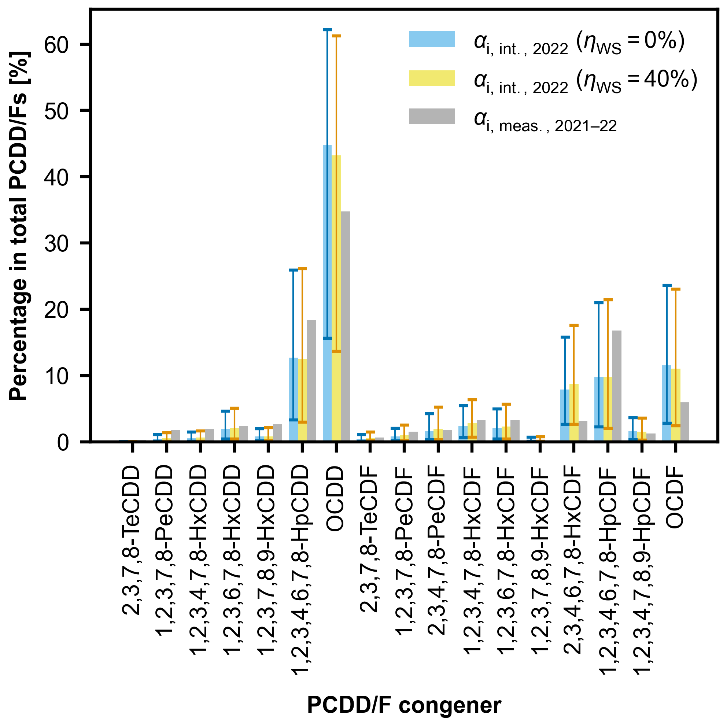
**

**Fig. S4**: Model-derived soil profile for 2022 (blue and yellow bars) and measurement-derived soil profile for 2021–2022 (gray bars), referring to the Vallon MSWI pollution case. Soil profile simulated under two WS efficiency scenarios for PCDD/F total emissions ($\eta_{\mathrm{WS}}$ = 0%, blue bars and $\eta_{\mathrm{WS}}$ = 40%, yellow bars). Uncertainty intervals represent the range between the 2.5% and 97.5% quantiles of the congener fractions, obtained from 1,000,000 random samples of the PCDD/F congener’s soil half-lives, varied from −50% to +100% of the reference values

Taking into account only the indicative uncertainty on the half-lives is already enough to note that the profiles derived from the model contain globally the profiles derived from the measurements, both in the case of a high WS efficiency ($\eta_{\mathrm{WS}}$ = 0%) and a low WS efficiency scenario ($\eta_{\mathrm{WS}}$ = 40%). The model established in this project are therefore not rejected by this second validation analysis. On the other hand, it cannot be validated either due to the significant influence of the uncertainties that emerge. It should be noted that the magnitude of the uncertainties, which is already large because of all the assumptions and simplifications made in developing the profile and quantity model, is compounded by the validation method itself. Indeed, new uncertainties are introduced when evaluating the values of half-lives, the effect of temperature on the decay constants, the choice of reaction kinetics, the impact of soil sorption on the congener profile, etc.

1. VA3 third and last validation method consists of comparing the residual amount of PCDD/Fs present in Lausanne soil in 2022, as derived from the profile and quantity model (involving the same time integration of the model estimates as for VA2) and as derived from the spatial interpolation of the 2021–2022 soil measurements in the Lausanne region. The residual amount of PCDD/Fs in soil in 2022 (expressed in TEQ_WHO-2005_ or TEQ_WHO-2022_ units), as estimated from the model developed in this project and from measurements in Lausanne area, is compared for validation purposes.

The residual amount of PCDD/Fs in soil on a TEQ basis, as derived by the project model, is simply obtained by adding the products of the congener residual amounts estimated in VA2 with their WHO toxic equivalency factors. Again, two model-derived estimates are provided and correspond to the two WS efficiency scenarios that have been established in section 3.3 of the main script ($\eta_{\mathrm{WS}}$ = 0% and $\eta_{\mathrm{WS}}$ = 40%). This yields respectively 1,698 and 1,283 gTEQ _WHO-2022_ or 1,419 and 1,115 gTEQ _WHO-2005_. To estimate the residual amount of PCDD/Fs from the 124 soil measurements carried out between 2021 and 2022 by Impact-Concept SA (2022), a more complex method is applied. First, the congener concentrations (measured in ng/kg_dry mass_) are converted into toxic equivalent units (gTEQ/kg_dry mass_). For each measurement point, the PCDD/F concentration is then calculated in toxic equivalent units (gTEQ/kg_dry mass_) simply by summing those of the 17 congeners at that location. Since all 124 soil measurements are performed at depths of 0–5 or 0–20 cm, the average PCDD/F concentrations in the top 50 cm of soil, corresponding approximately to the extent of pollution in Lausanne (Airmes AG 2021), is then derived at each measurement point by applying an adjustment factor to the PCDD/F measured concentrations. These adjustment factors are taken from the Airmes report, considering the exponential method (Airmes AG 2021). Once the average PCDD/F concentrations over the first 50 centimeters of soil have been estimated at the 124 sampling points, a spatial interpolation is performed using a Delaunay triangulation. The latter allows to interpolate the average PCDD/F concentrations for the first 50 cm of soil over the whole measurement area with a pixel resolution of 1 m^2^ (see Fig. S5). For each pixel, the residual amount of PCDD/Fs is then estimated by multiplying the average concentration value for the first 50 cm (gTEQ/kg_dry mass_) by the approximate density of a dry soil (*ρ*_soil_ = 1,500 kg/m^3^), the approximate depth of the pollution (*d* = 0.5 m) and the surface area of the pixel (*A_p_* = 1 m^2^). Finally, the PCDD/F residual amounts of all pixels are summed over the whole interpolation area. This gives a total PCDD/F residual amount of 425 gTEQ_WHO-2022_ or 371 gTEQ_WHO-2005_.


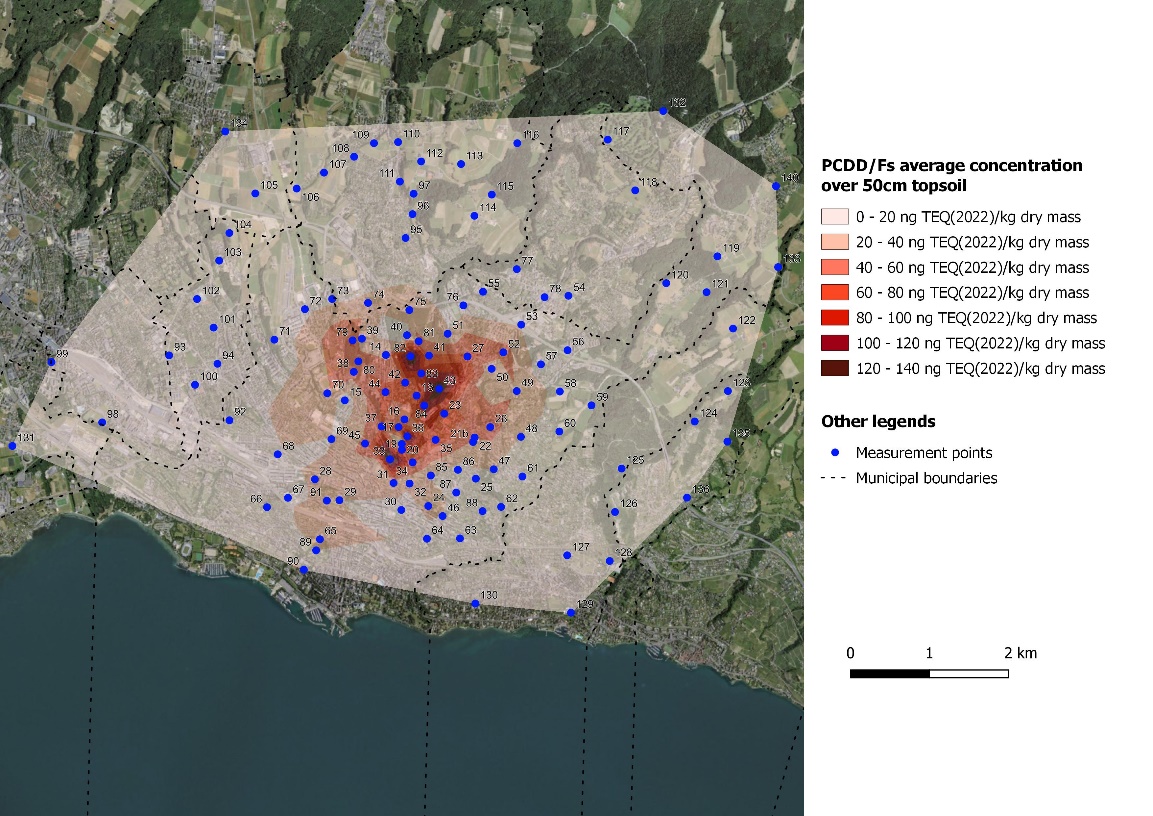


**Fig. S5** Delaunay interpolation of the average PCDD/F concentration for the first 50 cm of soil. Computed from 124 measurement points spread over Lausanne region. Resolution of 1 m^2^. Results displayed in terms of WHO-2022 toxicity

The value of 1,698 gTEQ_WHO-2022_ and 1,419 gTEQ_WHO-2005_ ($\eta_{\mathrm{WS}}$ = 0%) or 1,283 gTEQ_WHO-2022_ and 1,115 gTEQ_WHO-2005_ ($\eta_{\mathrm{WS}}$ = 40%) estimated from the project model is in the same order of magnitude as the 425 gTEQ_WHO-2022_ and 371 gTEQ_WHO-2005_ deduced from the soil measurements. A gross error in the methodology used to develop the emission profile and quantity model is thus a priori excluded.

Nevertheless, there appears to be a significant overestimation of the estimated amount of PCDD/Fs deposited in the measurement area. This was, however, expected and is attributable to the simplifications of the comparative calculation applied. In particular, it was assumed here that all modeled PCDD/F emissions were deposited in the measurement area and remained there over time. In reality, a part was transported by air outside the perimeter and a part that was deposited there flowed out of the perimeter in the course of time due to physico-chemical mechanisms, like run-out. The amount of residual PCDD/Fs deposited in soil according to the project model is therefore systematically overestimated. Conversely, in the case of the estimation of the residual PCDD/F amount in soil from the measurements, the topography was not taken into account when interpolating (flat surface assumed). This leads to an underestimation of the area of the measurement perimeter and thus of the amount of PCDD/Fs deposited. To another extent, the inherent uncertainties in the developed model and measurements also explain the discrepancies between model-derived and measurement-derived soil quantities. The comparative method, in addition to the systematic biases discussed above, introduces further uncertainties as well (related to the interpolation method, reaction kinetics, dry soil density, half-lives, pollution depth, adjustment factors applied, etc.).

# References

Airmes AG (2021) Expertise on the origin of soil pollution in Lausanne. Tech. Rep. No. 7300, 32 pp. (in French)

Bunsan S, Chen W-Y, Chen H-W, et al (2013) Modeling the dioxin emission of a municipal solid waste incinerator using neural networks. Chemosphere 92:258–264. https://doi.org/10.1016/j.chemosphere.2013.01.083

Chang MB, Chi KH, Chang-Chien GP (2004) Evaluation of PCDD/F congener distributions in MWI flue gas treated with SCR catalysts. Chemosphere 55:1457–1467. https://doi.org/10.1016/j.chemosphere.2004.01.005

Bulletins du Conseil communal - Secrétariat du Conseil communal de Lausanne (1952–2005) Bulletins of the Communal Council of Lausanne (in French)

DeVito M, Bokkers B, van Duursen MBM, et al (2024) The 2022 world health organization reevaluation of human and mammalian toxic equivalency factors for polychlorinated dioxins, dibenzofurans and biphenyls. Regulatory Toxicology and Pharmacology 146:105525. https://doi.org/10.1016/j.yrtph.2023.105525

Direction générale de l’environnement (DGE), Canton de Vaud (2021) Limit values for the valorization (OSol) and landfilling (OLED) of earth and excavation materials from construction works, DCPE 877 (in French)

eOde (2022) Soil pollution in Lausanne with dioxins and furans - Identification of possible pollution sources through multivariate statistical analysis of available data. Final Report, 38 pp. (in French)

Etat de Vaud (2021) Press release - Discovery of past soil pollution in Lausanne (in French)

Everaert K, Baeyens J (2002) The formation and emission of dioxins in large scale thermal processes. Chemosphere 46:439–448. https://doi.org/10.1016/S0045-6535(01)00143-6

Impact-Concept SA (2022) Evaluation of soil pollution by dioxins and furans. Tech. Rep. No. 1877-RA-03, 355 pp. (in French)

Isler J (1999) Garbage breakdown and foul odors. 24heures (in French)

Jeanneret P (1969) On the sidelines of the lost packaging survey. Bulletin de l'Association Romande pour la Protection des Eaux et de l'Air (ARPEA), 32, 46–49 (in French)

Jones KC, Duarte-Davidson R (1997) Transfers of airborne PCDD/Fs to bulk deposition collectors and herbage. Environ Sci Technol 31:2937–2943. https://doi.org/10.1021/es970133t

Kilgroe JD (1996) Control of dioxin, furan, and mercury emissions from municipal waste combustors. J Hazard Mater 47:163–194. https://doi.org/10.1016/0304-3894(95)00108-5

Li CS, Jenq FT (1993) Physical and chemical composition of hospital waste. Infect Control Hosp Epidemiol 14:145–150. https://doi.org/10.1086/646700

Liu F, Liu H-Q, Wei G-X, et al (2018) Characteristics and treatment methods of medical waste incinerator fly ash: A review. Processes 6:173. https://doi.org/10.3390/pr6100173

Luo Y, Yang X (2007) A multimedia environmental model of chemical distribution: Fate, transport, and uncertainty analysis. Chemosphere 66:1396–1407. https://doi.org/10.1016/j.chemosphere.2006.09.026

MacLeod M, Fraser AJ, Mackay D (2002) Evaluating and expressing the propagation of uncertainty in chemical fate and bioaccumulation models. Environ Toxicol Chem 21:700–709. https://doi.org/10.1002/etc.5620210403

McLachlan MS, Sewart AP, Bacon JR, Jones KC (1996) Persistence of PCDD/Fs in a sludge-amended soil. Environ Sci Technol 30:2567–2571. https://doi.org/10.1021/es950932g

Municipalité de Lausanne (1958) Annual Management Reports (in French)

Paasivirta J, Sinkkonen S, Mikkelson P, et al (1999) Estimation of vapor pressures, solubilities and Henry’s law constants of selected persistent organic pollutants as functions of temperature. Chemosphere 39:811–832. https://doi.org/10.1016/S0045-6535(99)00016-8

Peng Y, Lu S, Li X, et al (2020) Formation, measurement, and control of dioxins from the incineration of municipal solid wastes: Recent advances and perspectives. Energy Fuels 34:13247–13267. https://doi.org/10.1021/acs.energyfuels.0c02446

Quinche J-P (1983) Contamination of tree foliage by lead and mercury in the vicinity of a municipal waste incineration plant. Bulletin de l'Association Romande pour la Protection des Eaux et de l'Air (ARPEA), 117, 36–41 (in French)

Quinche J-P (1984) The contamination of soil by heavy metals in the vicinity of a municipal waste incineration plant. Bulletin de l'Association Romande pour la Protection des Eaux et de l'Air (ARPEA), 122, 22–27 (in French)

Riber C, Petersen C, Christensen TH (2009) Chemical composition of material fractions in Danish household waste. Waste Manag 29:1251–1257. https://doi.org/10.1016/j.wasman.2008.09.013

Rollier H, Schwab C, Völgyi G (1983) Heavy metal removal during wet scrubbing of exhaust gas in the waste incineration plant in Lausanne. Müll und Abfall 6/83:158–164 (in German)

Schroy JM, Hileman FD, Cheng S (1985) Physical/chemical properties of 2, 3, 7, 8-tetrachlorodibenzo-p-dioxin. In: Aquatic Toxicology and Hazard Assessment: Eighth Symposium. American Society for Testing and Materials, pp 409–421

Seike N, Kashiwagi N, Otani T (2007) PCDD/F contamination over time in Japanese paddy soils. Environ Sci Technol 41:2210–2215. https://doi.org/10.1021/es062318i

Sinkkonen S, Paasivirta J (2000) Degradation half-life times of PCDDs, PCDFs and PCBs for environmental fate modeling. Chemosphere 40:943–949. https://doi.org/10.1016/S0045-6535(99)00337-9

Stanmore BR (2004) The formation of dioxins in combustion systems. Combust Flame 136:398–427. https://doi.org/10.1016/j.combustflame.2003.11.004

Syfrig M (1958) The purifying fire - Lausanne leaves no trace of its waste burned at nearly 1000°C. Tribune de Lausanne (in French)

Takaoka M, Liao P, Takeda N, et al (2003) The behavior of PCDD/Fs, PCBs, chlorobenzenes and chlorophenols in wet scrubbing system of municipal solid waste incinerator. Chemosphere 53:153–161. https://doi.org/10.1016/S0045-6535(03)00437-5

Themelis NJ (2010) Chlorine Sources, Sinks, and Impacts in WTE Power Plants. In: North American Waste-to-Energy Conference. American Society of Mechanical Engineers Digital Collection, pp 77–84. https://doi.org/10.1115/NAWTEC18-3577

van den Berg M, Birnbaum LS, Denison M, et al (2006) The 2005 World Health Organization re-evaluation of human and mammalian toxic equivalency factors for dioxins and dioxin-like compounds. Toxicol Sci 93:223–241. https://doi.org/10.1093/toxsci/kfl055

Voelgyi G (1985) Thermal recovery of municipal waste and flue gas treatment at the Lausanne household waste incineration plant. VDI Berichte, 204–222 (in French)

Wallenhorst T (1996) Investigations into the distribution and airborne transport of polychlorinated dibenzo-*p*-dioxins and dibenzofurans in Baden-Württemberg. Eberhard-Karls-Universität zu Tübingen

Wang L, Lee W, Lee W, et al (2003) Effect of chlorine content in feeding wastes of incineration on the emission of polychlorinated dibenzo-*p*-dioxins/dibenzofurans. Sci Total Environ 302:185–198. https://doi.org/10.1016/S0048-9697(02)00306-6

Weber R, Hagenmaier H (1999) Mechanism of the formation of polychlorinated dibenzo-*p*-dioxins and dibenzofurans from chlorophenols in gas phase reactions. Chemosphere 38:529–549. https://doi.org/10.1016/S0045-6535(98)00200-8

White HJ (1957) Fifty years of electrostatic precipitation. J Air Pollut Control Assoc 7:166–177. https://doi.org/10.1080/00966665.1957.10467797

Zar JH (1972) Significance testing of the Spearman rank correlation coefficient. J Am Stat Assoc 67:578–580. https://doi.org/10.2307/2284441
